# Supplementary material for: Construction of Benzenesulfonamide Derivatives via Copper and Visible Light-induced Azides and S(O)2–H Coupling
Source: Molecules. 2022 Aug 28;27(17):5539. doi: 10.3390/molecules27175539 (PMC9457716; doi:10.3390/molecules27175539)

Supporting Information

Construction of Benzenesulfonamide Derivatives via Copper and Visible Light-induced Azides and S(O)2–H Coupling

Zhipeng Liang, Ya-Nan Wu* and Yang Wang*

School of chemistry and chemical engineering, Nantong University

E-mail: wuyanan@ntu.edu.cn

Table of Contents

General Information…………………………………………………………...2

General Procedure for Preparation of 3 ………………………….…..……2-7

^{1}H&^{13}C NMR Spectra ……………………………………………………….8-23

General Information

All reactions and manipulations involving air- or moisture-sensitive compounds were performed using standard Schlenk techniques. Solvents were purified and dried according to standard methods prior to use. NMR spectras were recorded on a Bruker ARX 400 spectrometer and were recorded in ppm (δ) downfield of TMS (δ = 0) in deuterated solvent. Signal splitting patterns are described as singlet (s), doublet (d), triplet (t), quartet (q), quintet (quint), or multiplet (m), with coupling constants (J) in hertz. Mass spectra were conducted at Micromass Q-Tof instrument (ESI) and Agilent Technologies 5973N (EI).

General procedure for the synthesis of products 3

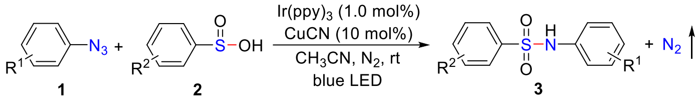

Figure S1 The procedure for benzenesulfonamide derivatives synthesis.

Arylsulfinic acid **2** (0.10 mmol), Ir(ppy)$_3$ (1.0 mol%) and CuCN (10 mol%) were added to a 10 mL schlenk tube. Under the protection of N_2, azide (0.11 mmol) and CH_3CN (1.0 mL) were then dropped to the schlenk tube equipped with a stirring bar. The solution was stirred at a distance of 1.5 cm from a 24 W blue LED lamp at room temperature for 24 h. The reaction system was extracted with EtOAc (3 x 10 mL), washed with saturated NaCl solution (10 mL), dried over Na_2SO_4, and concentrated the organic layer under reduced pressure. The product was purified by flash chromatography.

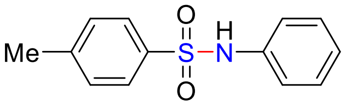

Figure S2 4-methyl-*N*-phenylbenzenesulfonamide (3a)

TLC R$_f$ = 0.5 (PE/EA = 3:1); 15.1 mg, 61% yield; white solid; ^{1}H NMR (400 MHz, $CDCl_3$) δ 7.66 – 7.64 (m, 2H), 7.26 – 7.21 (m, 4H), 7.13 – 7.04 (m, 3H), 6.64 (s, 1H), 2.23 (s, 3H); ^{13}C NMR (101 MHz, $CDCl_3$) δ 143.9, 136.4, 136.0, 129.6, 129.3, 127.3, 125.4, 121.7, 21.6; HRMS (ESI) ([M+H]$^+$) Calcd. For $C_{13}H_{14}NO_2S$: 248.0740, found: 248.0737.

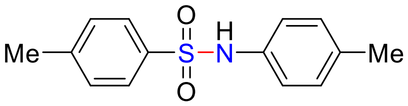

Figure S3 4-methyl-*N*-(*p*-tolyl)benzenesulfonamide (3b)

TLC R_f = 0.5 (PE/EA = 3:1); 22.4 mg, 86% yield; white solid; ^{1}H NMR (400 MHz, $CDCl_3$) δ 7.66 – 7.64 (m, 2H), 7.22 – 7.20 (m, 2H), 7.03 – 7.01 (m, 2H), 6.97 – 6.95 (m, 3H), 2.37 (s, 3H), 2.26 (s, 3H); ^{13}C NMR (101 MHz, $CDCl_3$) δ 143.7, 136.0, 135.3, 133.8, 129.8, 129.6, 127.3, 122.2, 21.5, 20.8; HRMS (ESI) ($[M+H]^+$) Calcd. For $C_{14}H_{16}NO_2S$: 262.0896, found: 262.0897.

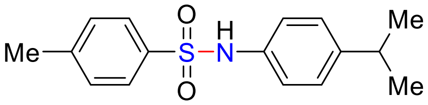

Figure S4 *N*-(4-isopropylphenyl)-4-methylbenzenesulfonamide (3c)

TLC R_f = 0.5 (PE/EA = 3:1); 23.7 mg, 82% yield; white solid; ^{1}H NMR (400 MHz, $CDCl_3$) δ 7.65 – 7.63 (m, 2H), 7.23 – 7.21 (m, 2H), 7.10 – 7.08 (m, 2H), 6.98 – 6.96 (m, 2H), 6.56 (s, 1H), 2.83 (m, 1H), 2.38 (s, 3H), 1.19 (d, J = 8.0 Hz, 6H); ^{13}C NMR (101 MHz, $CDCl_3$) δ 146.3, 143.7, 136.3, 134.0, 129.7, 129.6, 127.3, 127.2, 127.0, 122.2, 33.5, 23.9, 21.6; HRMS (ESI) ($[M+H]^+$) Calcd. For $C_{16}H_{20}NO_2S$: 290.1209, found: 290.1206.

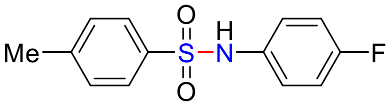

Figure S5 *N*-(4-fluorophenyl)-4-methylbenzenesulfonamide (3d)

TLC R_f = 0.5 (PE/EA = 3:1); 20.7 mg, 78% yield; white solid; ^{1}H NMR (400 MHz, $CDCl_3$) δ 7.56 – 7.54 (m, 2H), 7.18 – 7.15 (m, 2H), 6.99 – 6.92 (m, 2H), 6.88 – 6.82 (m, 3H), 2.31 (s, 3H); ^{13}C NMR (101 MHz, $CDCl_3$) δ 159.6 (d, J = 242.4 Hz), 143.0, 134.6, 131.2 (d, J = 3.0 Hz), 128.7, 126.2, 123.6 (d, J = 8.1 Hz), 115.0 (d, J = 23.2 Hz), 20.5; ^{19}F NMR (376 MHz, $CDCl_3$) δ -116.2; HRMS (ESI) ($[M+H]^+$) Calcd. For $C_{13}H_{13}FNO_2S$: 266.0646, found: 266.0648.

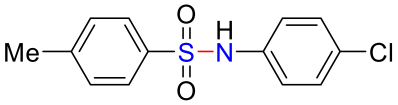

Figure S6 *N*-(4-chlorophenyl)-4-methylbenzenesulfonamide (3e)

TLC R_f = 0.5 (PE/EA = 3:1); 20.0 mg, 71% yield; white solid; ^{1}H NMR (400 MHz,

$CDCl_3$) δ 7.59 – 7.53 (m, 2H), 7.21 – 7.08 (m, 4H), 6.96 – 6.93 (m, 2H), 6.84 (s, 1H), 2.32 (s, 3H); ^{13}C NMR (101 MHz, $CDCl_3$) δ 143.2, 134.6, 134.0, 129.9, 128.8, 128.4, 126.2, 122.0, 20.5; HRMS (ESI) ($[M+H]^+$) Calcd. For $C_{13}H_{13}ClNO_2S$: 282.0350, found: 282.0346.

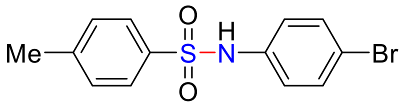

Figure S7 *N*-(4-bromophenyl)-4-methylbenzenesulfonamide (3f)

TLC R_f = 0.5 (PE/EA = 3:1); 23.7 mg, 73% yield; white solid; ^{1}H NMR (400 MHz, $CDCl_3$) δ 7.60 – 7.57 (m, 2H), 7.29 – 7.25 (m, 2H), 7.19 – 7.16 (m, 2H), 6.19 – 6.87 (m, 2H), 6.81 (s, 1H), 2.32 (s, 3H); ^{13}C NMR (101 MHz, $CDCl_3$) δ 143.2, 134.6, 134.6, 131.3, 128.8, 126.2, 122.1, 117.6, 20.6; HRMS (ESI) ($[M+H]^+$) Calcd. For $C_{13}H_{13}BrNO_2S$: 325.9845, found: 325.9848.

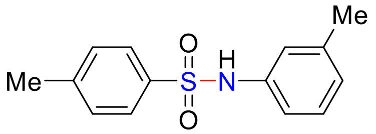

Figure S8 4-methyl-*N*-(*m*-tolyl)benzenesulfonamide (3g)

TLC R_f = 0.5 (PE/EA = 3:1); 21.4 mg, 82% yield; white solid; ^{1}H NMR (400 MHz, $CDCl_3$) 7.68 – 7.66 (m, 2H), 7.23 – 7.21 (m, 2H), 7.12 – 7.08 (s, 1H), 6.92 – 6.83 (m, 4H), 2.37 (s, 3H), 2.26 (s, 3H); ^{13}C NMR (101 MHz, $CDCl_3$) δ 143.8, 139.3, 136.4, 136.1, 129.6, 129.1, 127.3, 126.1, 122.1, 118.4, 21.6, 21.4; HRMS (ESI) ($[M+H]^+$) Calcd. For $C_{14}H_{16}NO_2S$: 262.0896, found: 262.0898.

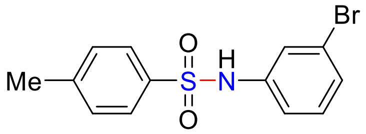

Figure S9 *N*-(3-bromophenyl)-4-methylbenzenesulfonamide (3h)

TLC R_f = 0.5 (PE/EA = 3:1); 23.3 mg, 72% yield; white solid; ^{1}H NMR (400 MHz, $CDCl_3$) δ 7.71 – 7.69 (m, 2H), 7.24 – 7.20 (m, 4H), 7.13 – 7.02 (m, 3H), 2.39 (s, 3H); ^{13}C NMR (101 MHz, $CDCl_3$) δ 144.3, 138.0, 135.7, 130.6, 129.8, 128.1, 127.3, 123.8, 122.8, 119.4, 21.6; HRMS (ESI) ($[M+H]^+$) Calcd. For $C_{13}H_{13}BrNO_2S$: 325.9845, found: 325.9844.

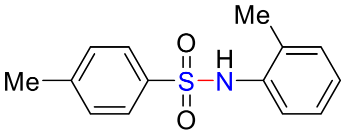

Figure S10 4-methyl-*N*-(*o*-tolyl)benzenesulfonamide (3i)

TLC R_f = 0.5 (PE/EA = 3:1); 19.3 mg, 74% yield; white solid; ^{1}H NMR (400 MHz, $CDCl_3$) δ 7.63 – 7.60 (m, 2H), 7.32 – 7.30 (m, 1H), 7.23 – 7.21 (m, 2H), 7.17 – 7.05 (m, 3H), 6.41 (s, 1H), 2.39 (s, 3H), 2.00 (s, 3H); ^{13}C NMR (101 MHz, $CDCl_3$) δ 143.8, 136.7, 134.5, 131.2, 130.8, 129.6, 127.2, 127.0, 126.2, 124.3, 21.6, 17.6; HRMS (ESI) ($[M+H]^+$) Calcd. For $C_{14}H_{16}NO_2S$: 262.0896, found: 262.0893.

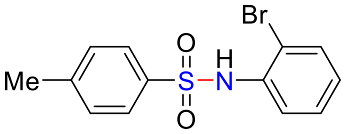

Figure S11 *N*-(2-bromophenyl)-4-methylbenzenesulfonamide (3j)

TLC R_f = 0.5 (PE/EA = 3:1); 22.7 mg, 70% yield; white solid; ^{1}H NMR (400 MHz, $CDCl_3$) δ 7.68 – 7.63 (m, 3H), 7.42 – 7.40 (m, 1H), 7.30 – 7.27 (m, 1H), 7.22 – 7.20 (m, 2H), 7.00 – 6.94 (m, 2H), 2.38 (s, 3H); ^{13}C NMR (101 MHz, $CDCl_3$) δ 144.2, 135.8, 134.7, 132.6, 129.6, 128.6, 127.3, 126.2, 122.5, 115.7, 21.6; HRMS (ESI) ($[M+H]^+$) Calcd. For $C_{13}H_{13}BrNO_2S$: 325.9845, found: 325.9844.

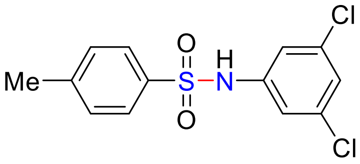

Figure S12 *N*-(3,5-dichlorophenyl)-4-methylbenzenesulfonamide (3k)

TLC R_f = 0.5 (PE/EA = 3:1); 18.2 mg, 58% yield; white solid; ^{1}H NMR (400 MHz, $CDCl_3$) δ 7.72 – 7.70 (m, 2H), 7.30 – 7.26 (m, 2H), 7.07 – 7.06 (m, 1H), 7.01 – 6.98 (m, 3H), 2.41 (s, 3H); ^{13}C NMR (101 MHz, $CDCl_3$) δ 144.7, 138.6, 135.6, 135.4, 130.0, 127.2, 125.0, 118.6, 21.6; HRMS (ESI) ($[M+H]^+$) Calcd. For $C_{13}H_{12}Cl_2NO_2S$: 315.9961, found: 315.9969.

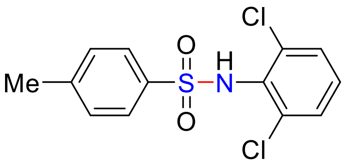

Figure S13 *N*-(2,6-dichlorophenyl)-4-methylbenzenesulfonamide (3l)

TLC R$_f$ = 0.5 (PE/EA = 3:1); 11.0 mg, 35% yield; white solid; ^{1}H NMR (400 MHz, $CDCl_3$) δ 7.64 – 7.62 (m, 2H), 7.27 –7.17 (m, 4H), 7.11 – 7.07 (m, 1H), 6.29 (s, 1H), 2.37 (s, 3H); ^{13}C NMR (101 MHz, $CDCl_3$) δ 143.0, 136.3, 134.0, 130.2, 128.5, 127.9, 127.8, 126.5, 20.6; HRMS (ESI) ([M+H]$^+$) Calcd. For $C_{13}H_{12}Cl_2NO_2S$: 315.9961, found: 315.9967.

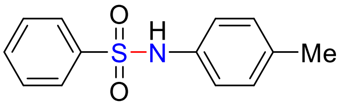

Figure S14 *N*-(*p*-tolyl)benzenesulfonamide (3m)

TLC R$_f$ = 0.5 (PE/EA = 3:1); 17.5 mg, 71% yield; white solid; ^{1}H NMR (400 MHz, $CDCl_3$) δ 7.76 – 7.74 (m, 2H), 7.53 (m, 1H), 7.45 – 7.43 (m, 2H), 7.04 – 7.02 (m, 2H), 6.96 – 6.93 (m, 2H), 6.62 (s, 1H), 2.27 (s, 3H); ^{13}C NMR (101 MHz, $CDCl_3$) δ 139.0, 135.7, 133.5, 132.9, 130.0, 129.0, 127.2, 122.6, 20.9; HRMS (ESI) ([M+H]$^+$) Calcd. For $C_{13}H_{14}NO_2S$: 248.0740,, found:248.0760.

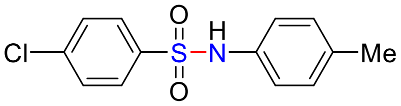

Figure S15 4-chloro-*N*-(*p*-tolyl)benzenesulfonamide (3n)

TLC R$_f$ = 0.5 (PE/EA = 3:1); 20.5 mg, 73% yield; white solid; ^{1}H NMR (400 MHz, $CDCl_3$) δ 7.69 – 7.66 (m, 2H), 7.40 – 7.37 (m, 2H), 7.10 –6.91 (m, 4H), 6.86 (s, 1H), 2.28 (s, 3H); ^{13}C NMR (101 MHz, $CDCl_3$) δ 139.4, 137.4, 136.0, 133.2, 130.0, 129.3, 128.7, 122.6, 20.9; HRMS (ESI) ([M+H]$^+$) Calcd. For $C_{13}H_{13}ClNO_2S$: 282.0350, found: 282.0345.

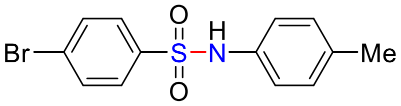

Figure S16 4-bromo-*N*-(*p*-tolyl)benzenesulfonamide (3o)

TLC R$_f$ = 0.5 (PE/EA = 3:1); 22.7 mg, 70% yield; white solid; ^{1}H NMR (400 MHz, $CDCl_3$) δ 7.63 – 7.55 (m, 4H), 7.13 –6.91 (m, 4H), 6.73 (s, 1H), 2.28 (s, 3H); ^{13}C

NMR (101 MHz, $CDCl_3$) δ 138.0, 136.0, 133.1, 132.2, 130.0, 128.8, 128.0, 122.7, 20.9; HRMS (ESI) ($[M+H]^+$) Calcd. For $C_{13}H_{13}BrNO_2S$: 325.9845, found: 325.9845.

Figure S17 The ^{1}H&^{13}C NMR Spectra of 3a-3o

4-methyl-*N*-phenylbenzenesulfonamide (3a)

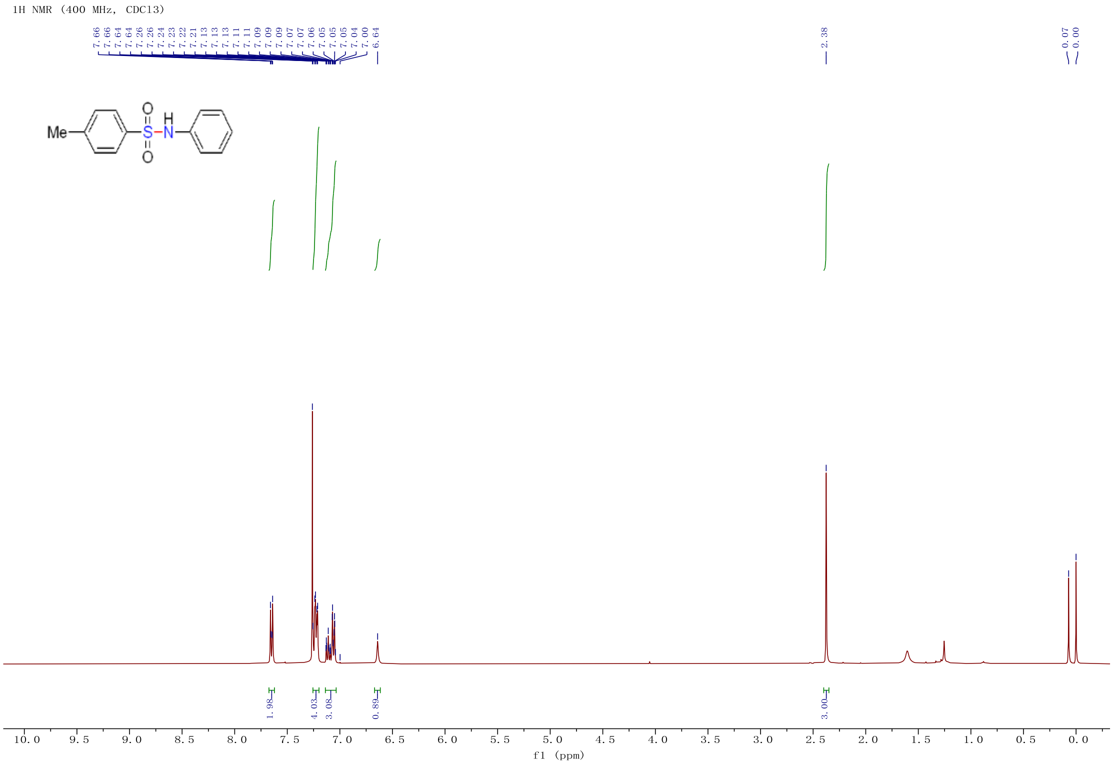

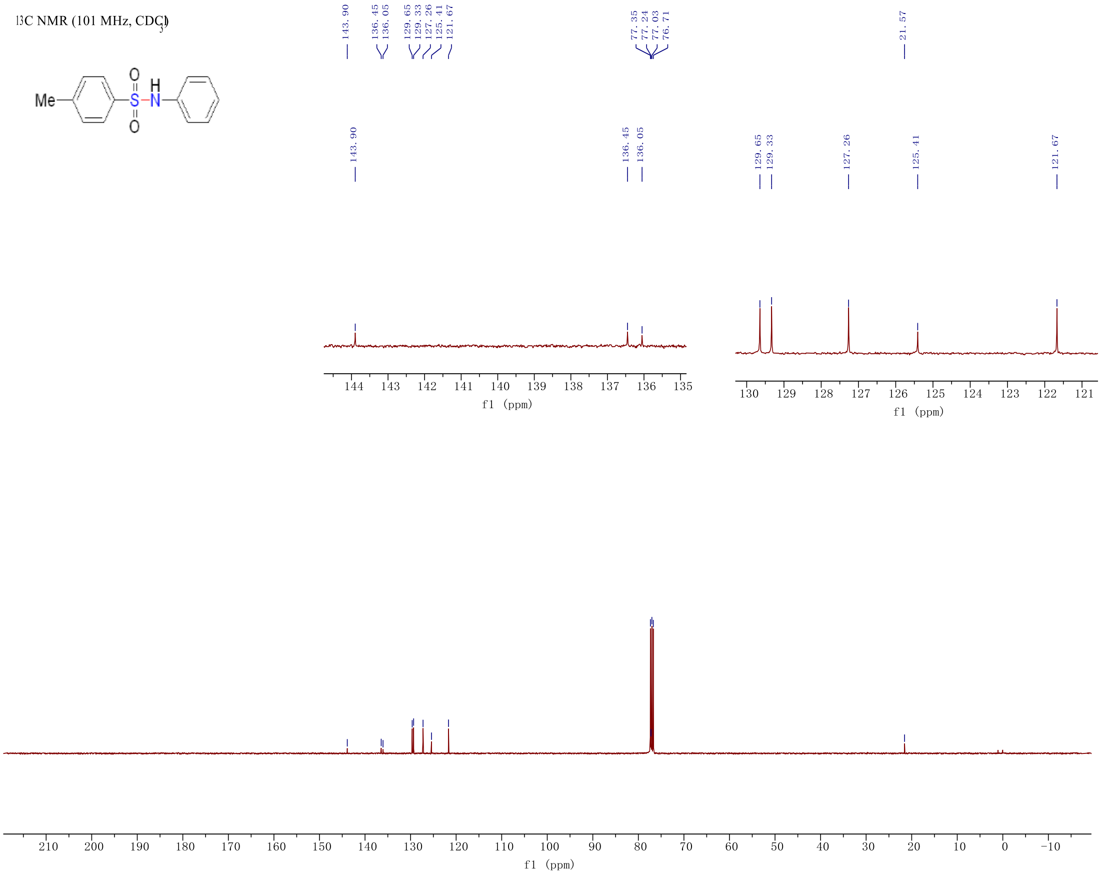

4-methyl-*N*-(*p*-tolyl)benzenesulfonamide (3b)

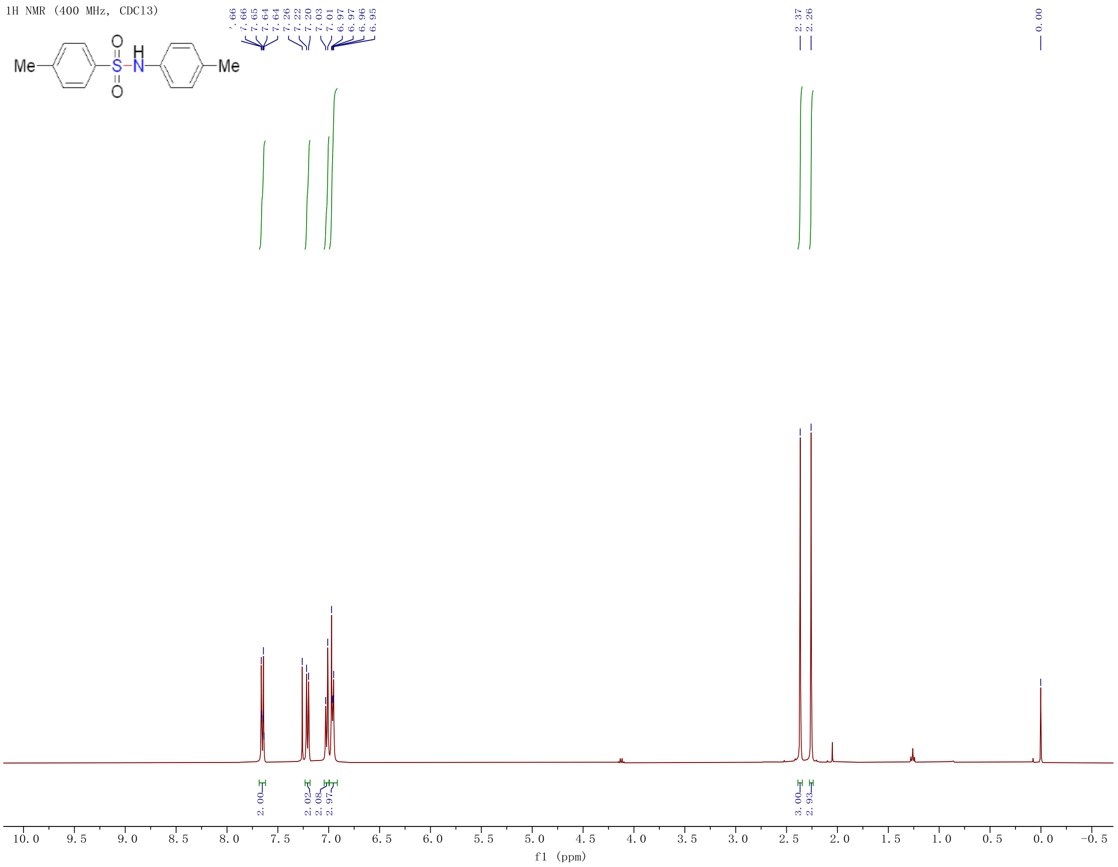

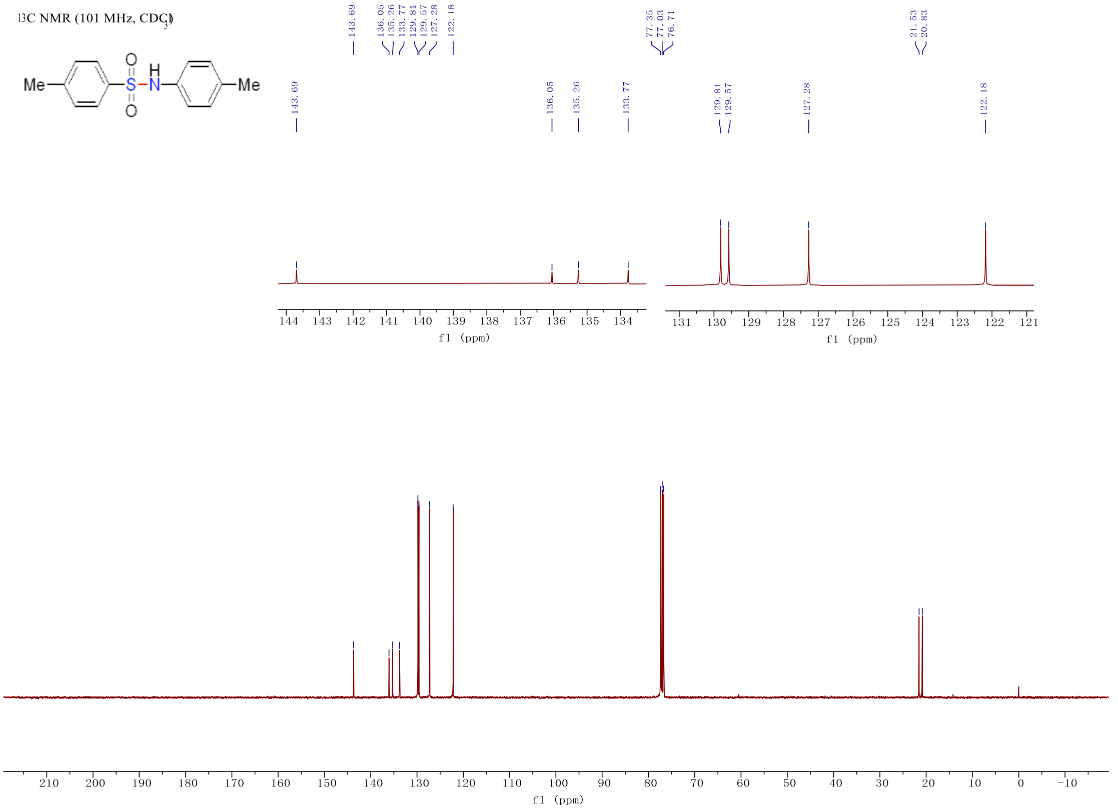

4-isopropyl-*N*-(*p*-tolyl)benzenesulfonamide (3c)

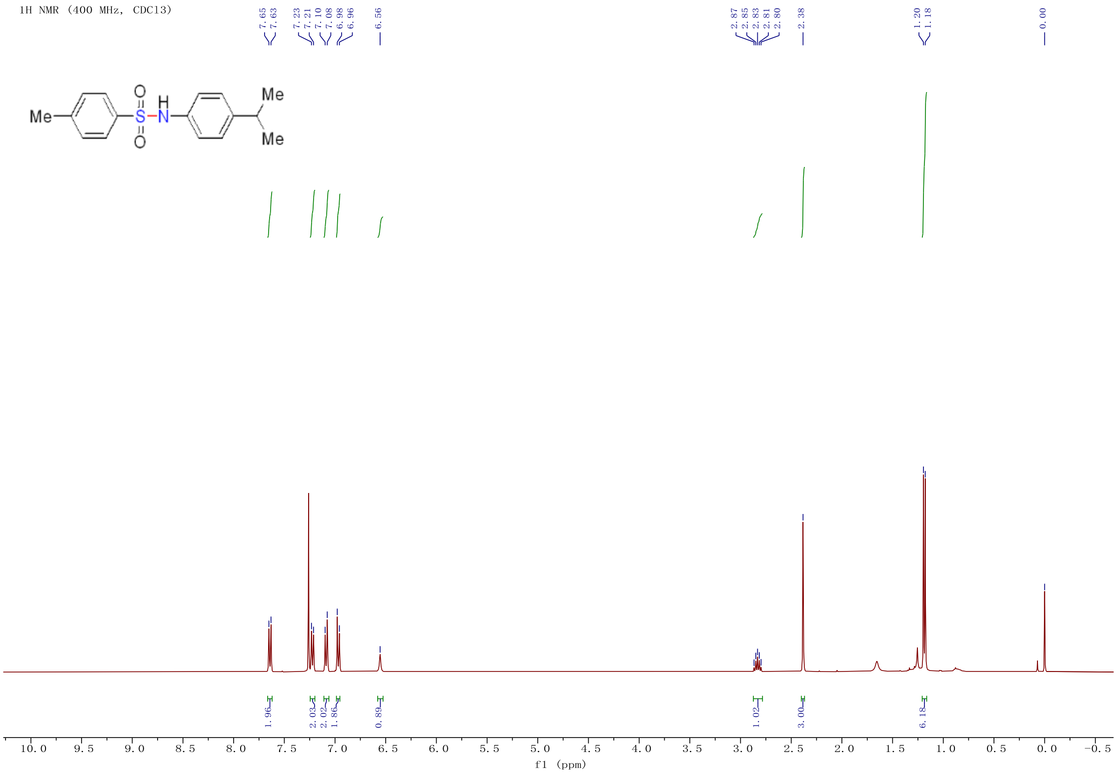

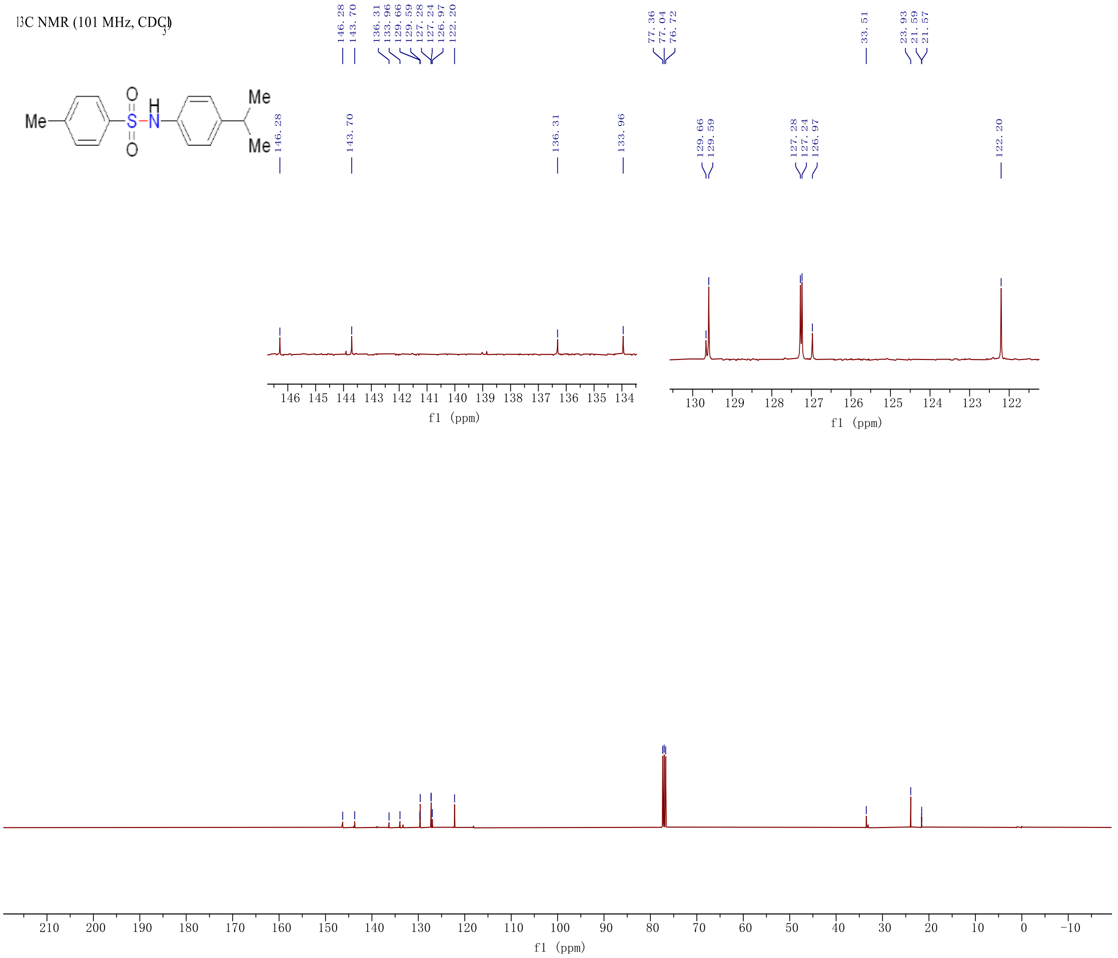

(4-fluorophenyl)-4-methylbenzenesulfonamide (3d)

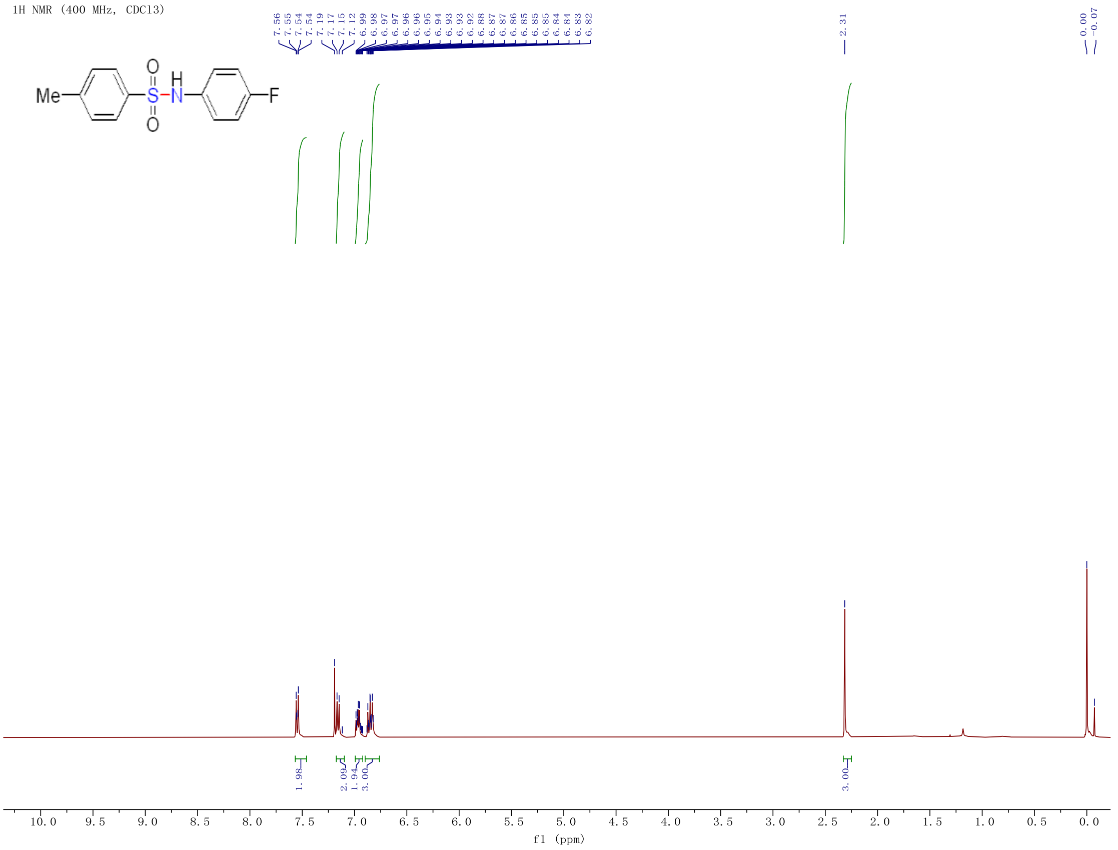

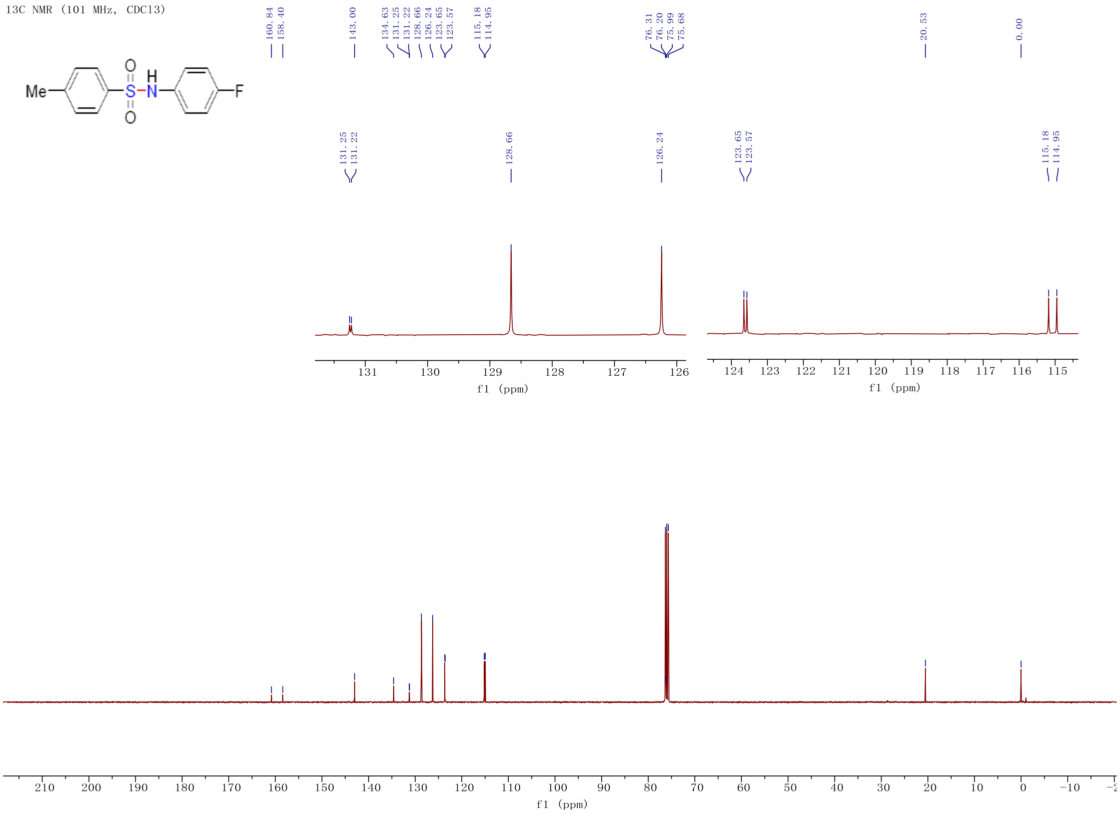

^{19}F NMR (376 MHz, $CDCl_3$) δ -116.24.

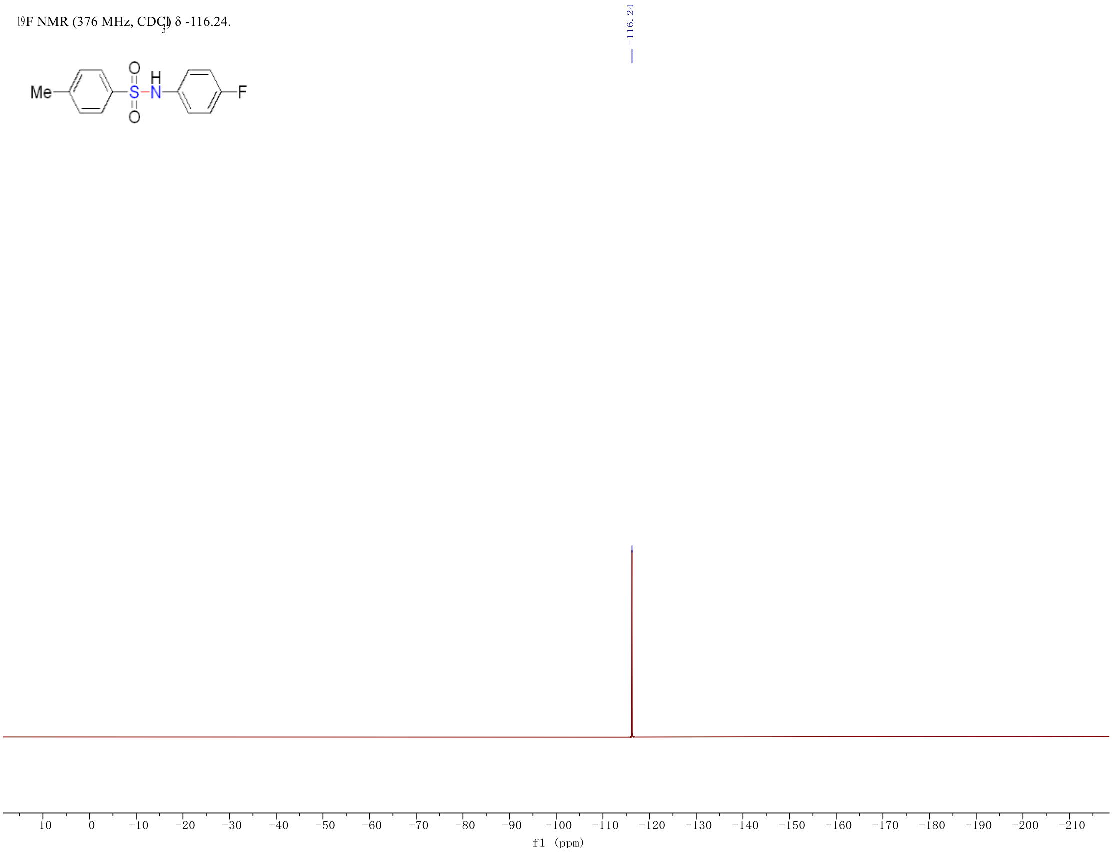

N-(4-chlorophenyl)-4-methylbenzenesulfonamide (3e)

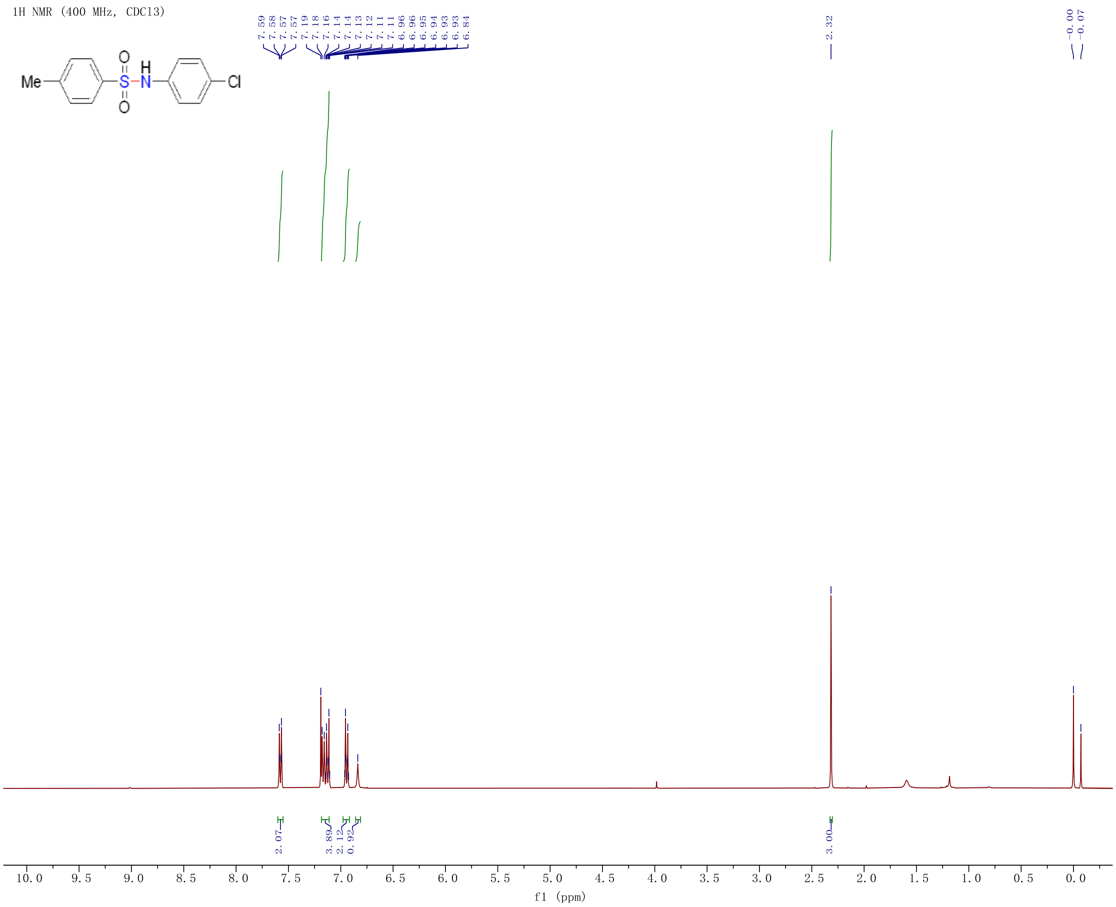

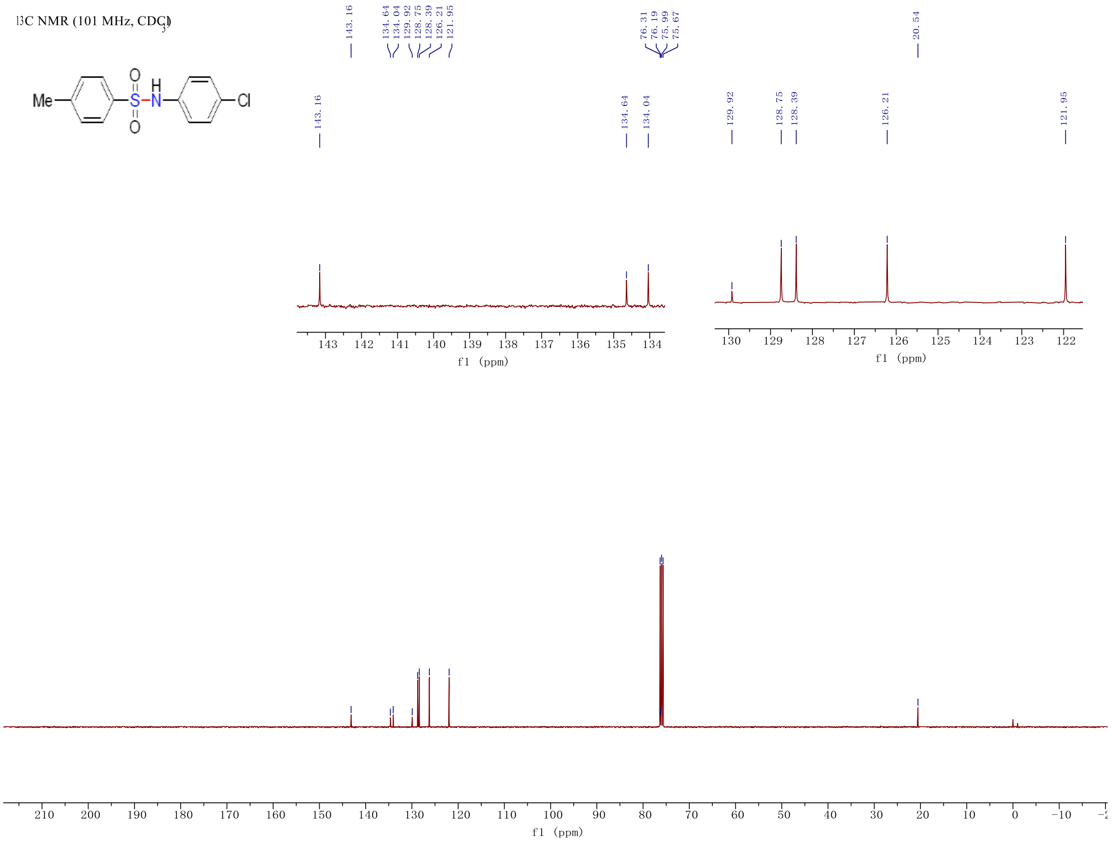

N-(4-bromophenyl)-4-methylbenzenesulfonamide (3f)

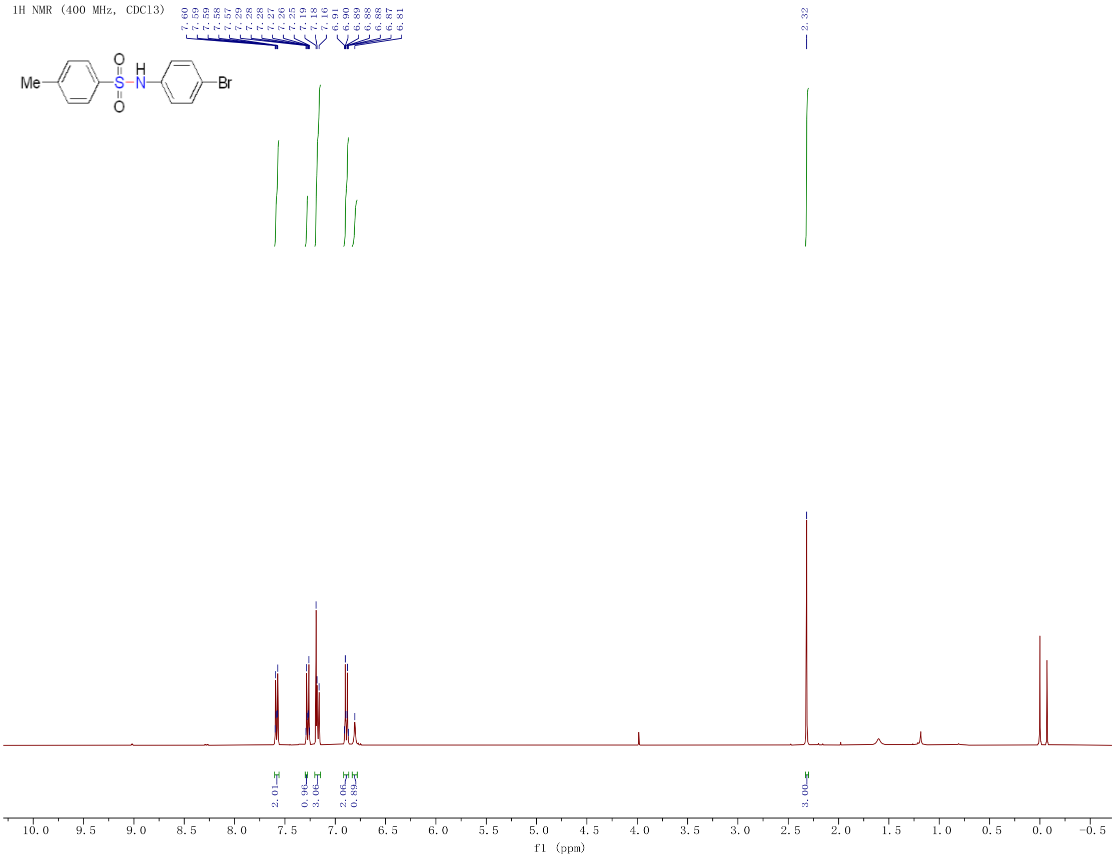

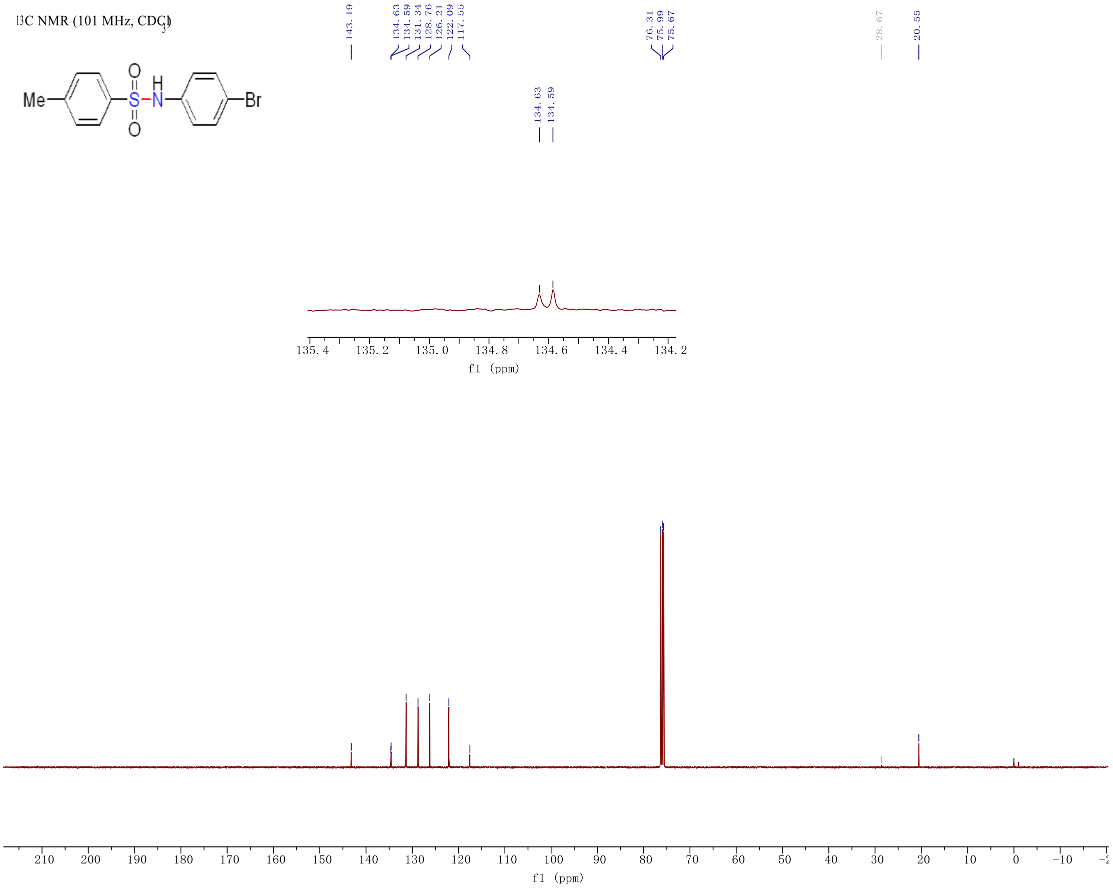

4-methyl-*N*-(*m*-tolyl)benzenesulfonamide (3g)

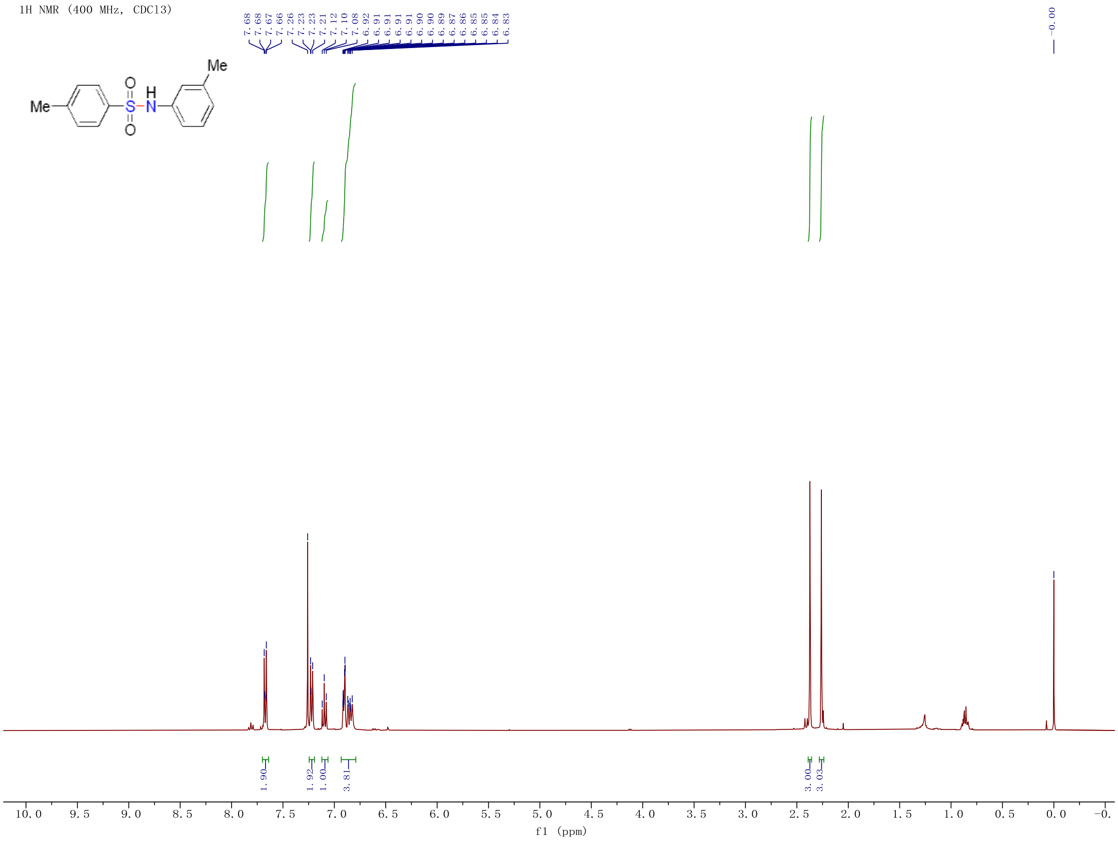

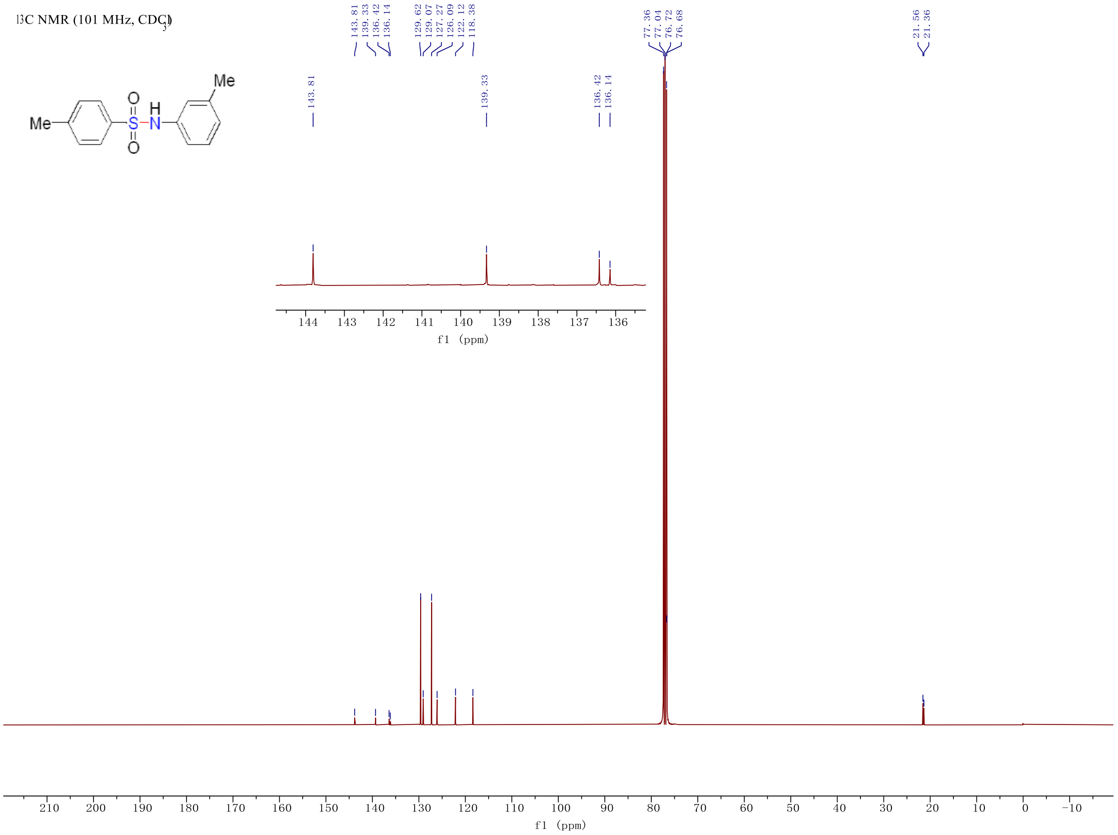

N-(4-bromophenyl)-4-methylbenzenesulfonamide (3h)

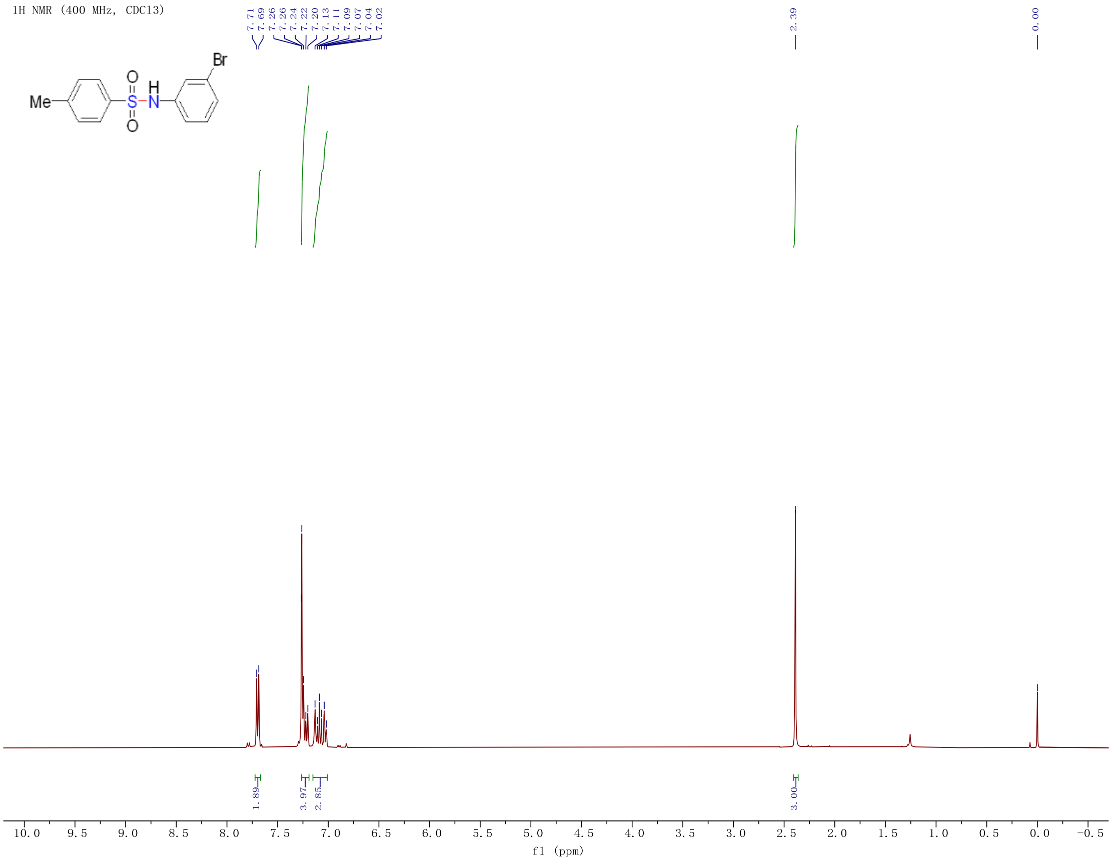

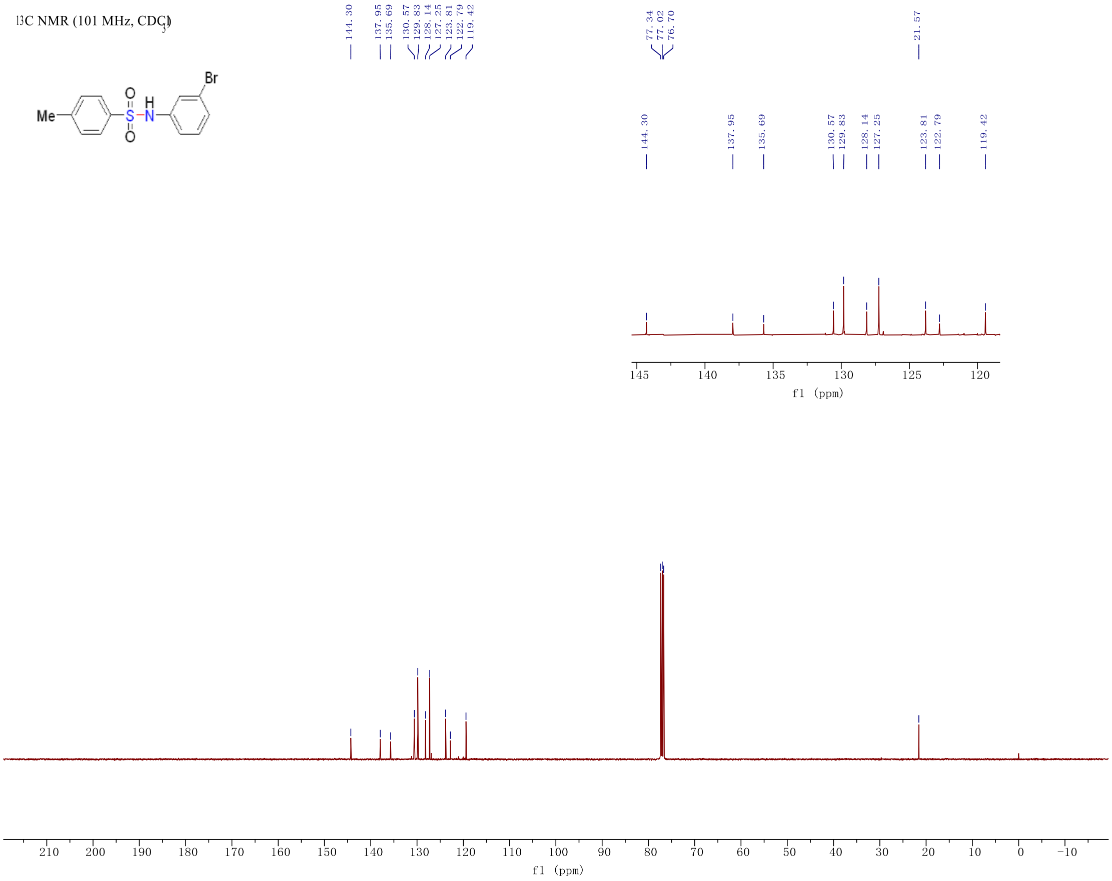

4-methyl-*N*-(*o*-tolyl)benzenesulfonamide (3i)

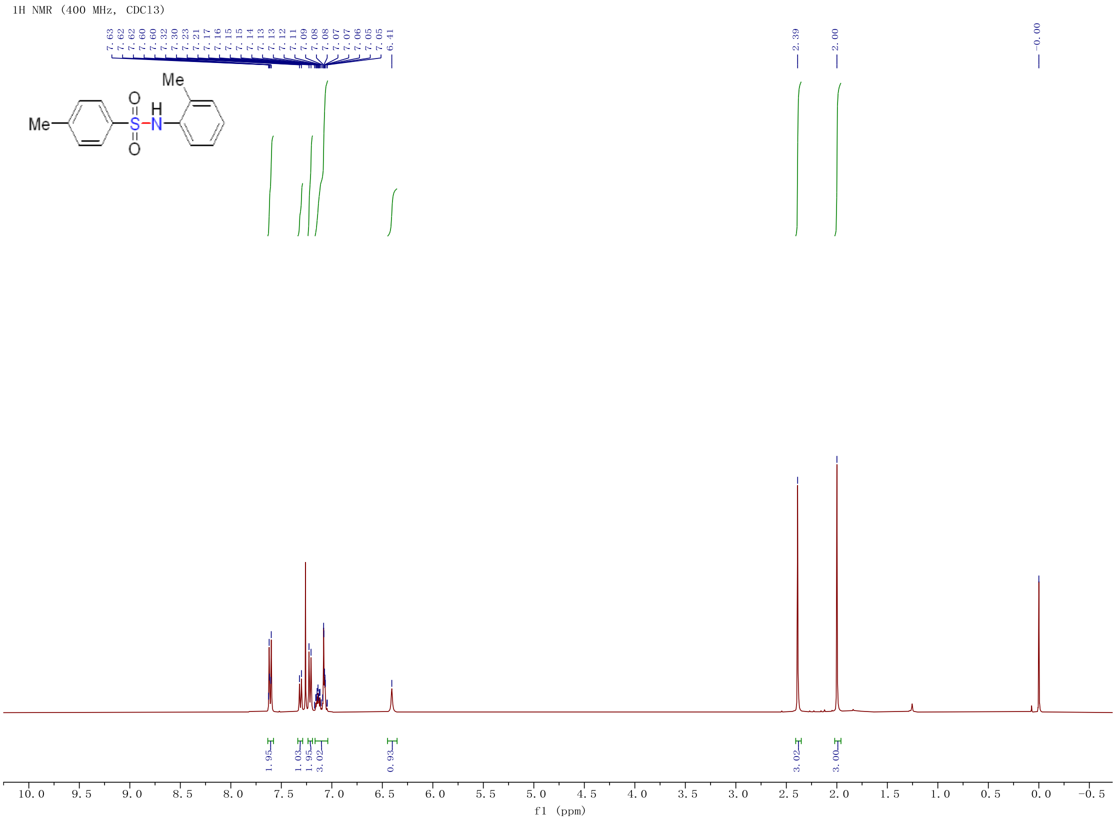

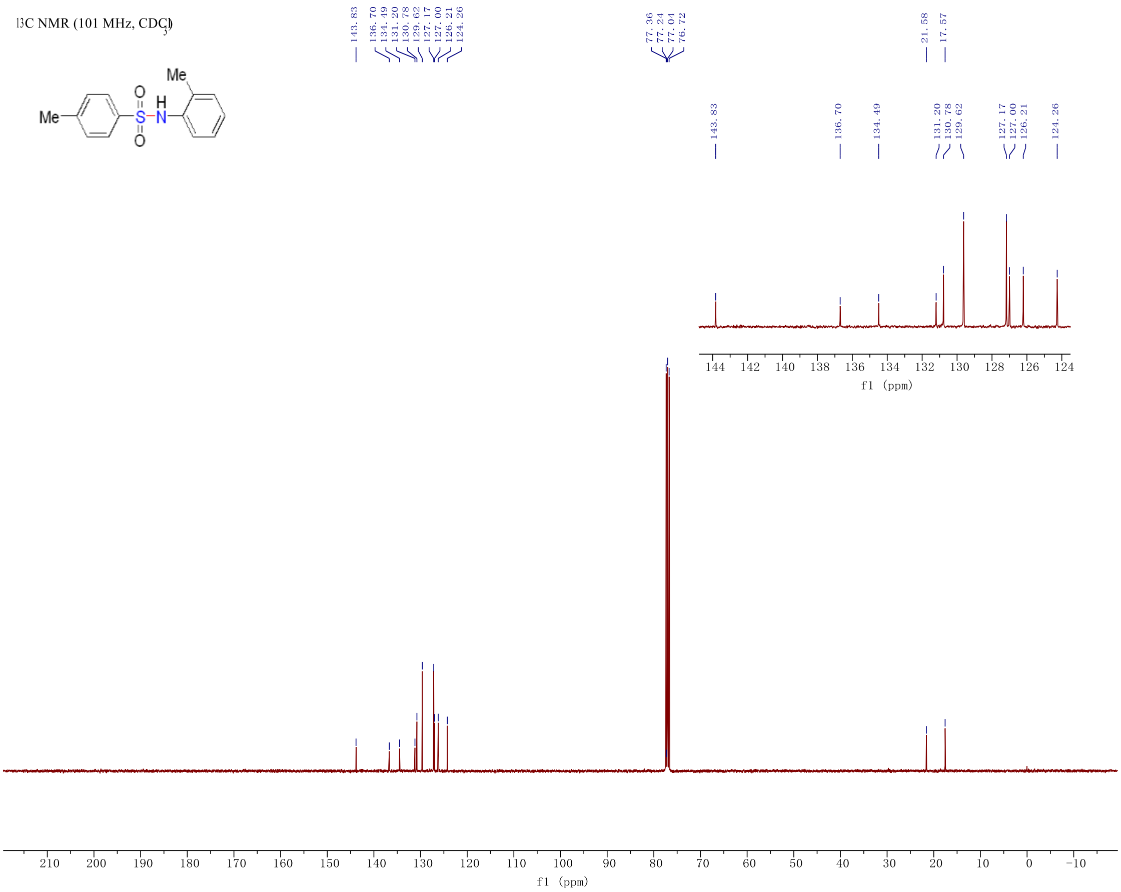

N-(2-bromophenyl)-4-methylbenzenesulfonamide (3j)

1H NMR (400 MHz, CDCl3)

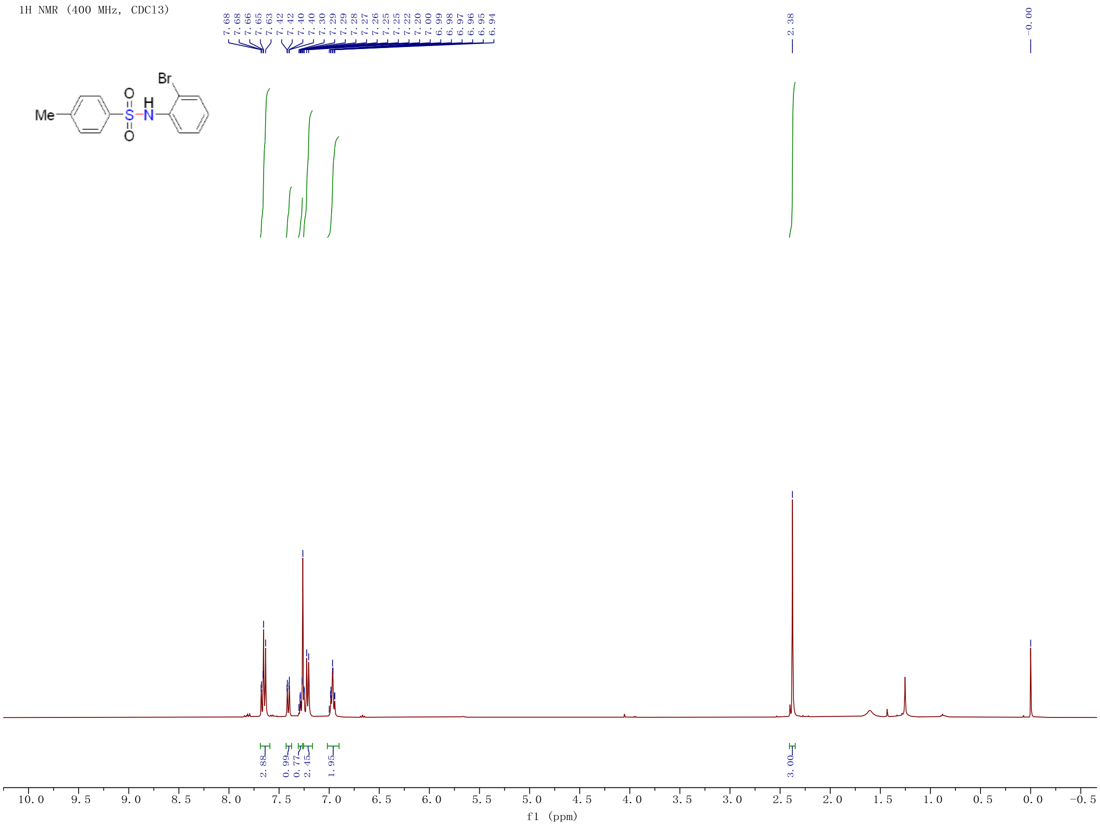

13C NMR (101 MHz, $CDCl_3$)

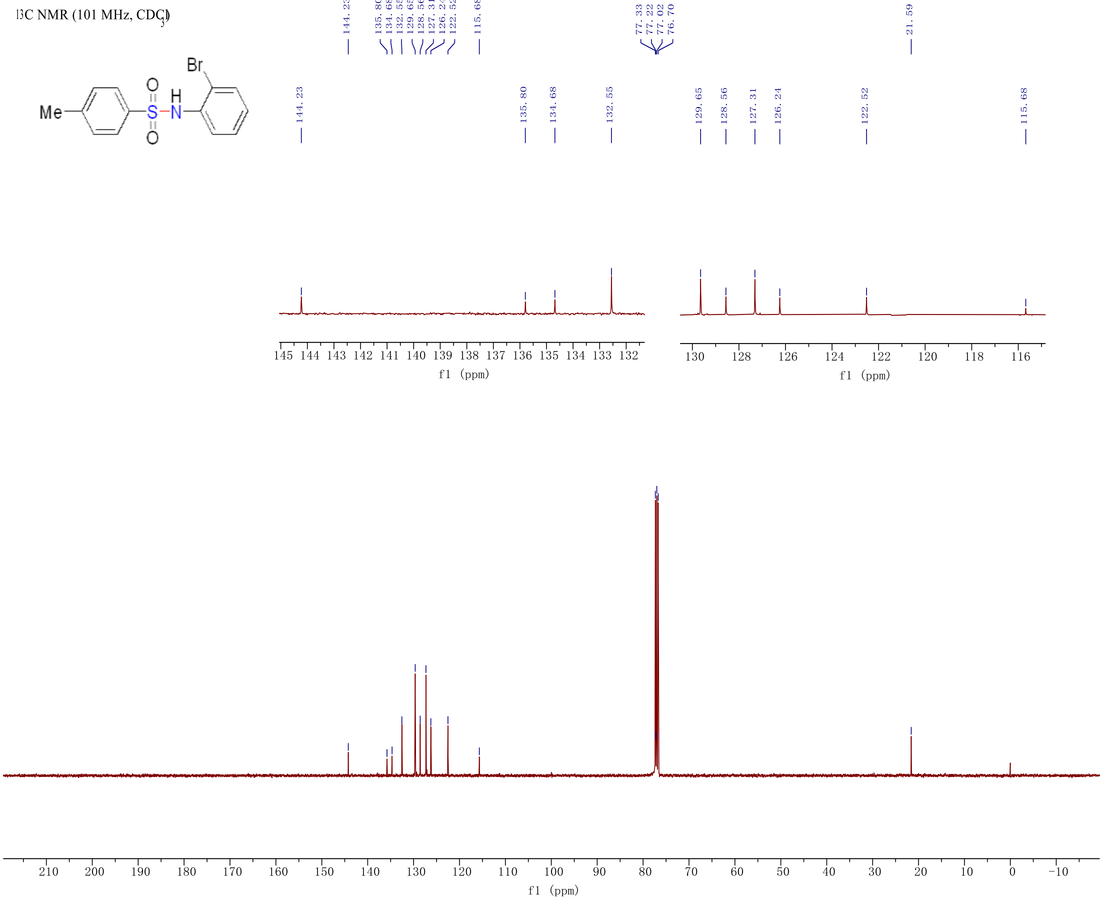

N-(3,5-dichlorophenyl)-4-methylbenzenesulfonamide (3k)

1H NMR (400 MHz, $CDCl_3$)

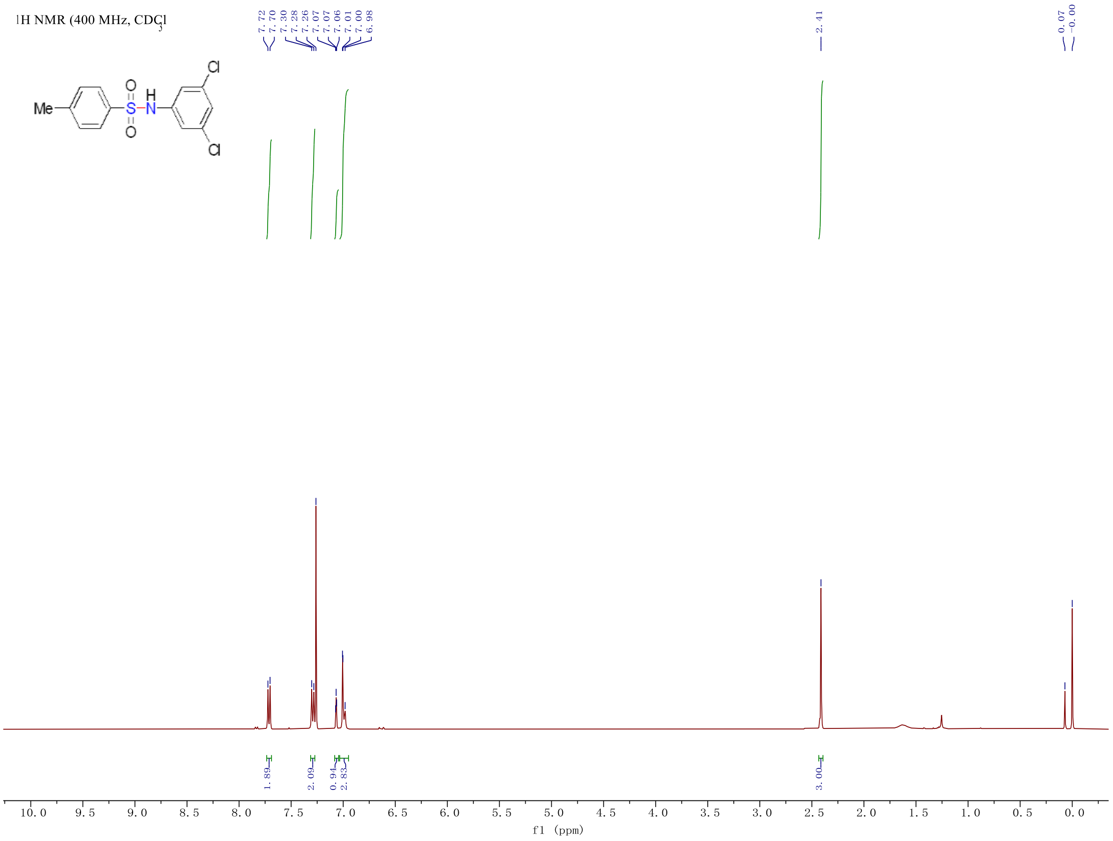

13C NMR (101 MHz, $CDCl_3$)

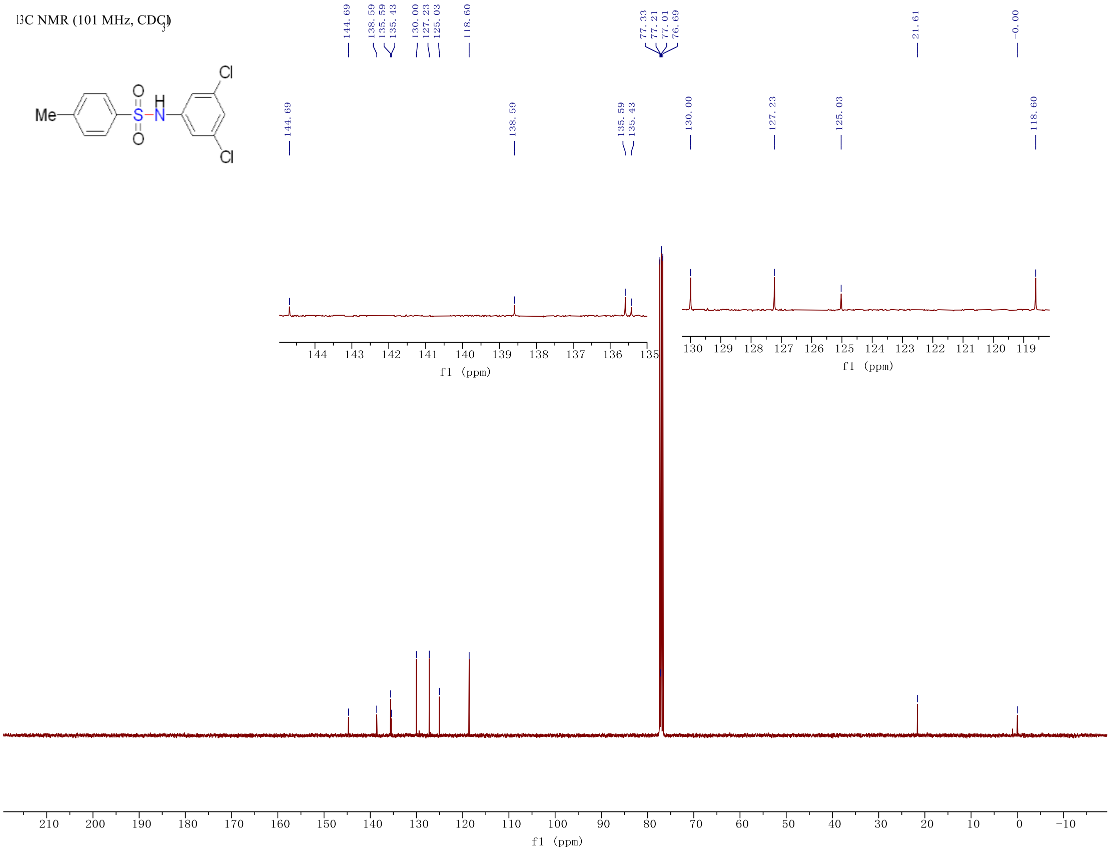

N-(2,6-dichlorophenyl)-4-methylbenzenesulfonamide (3l)

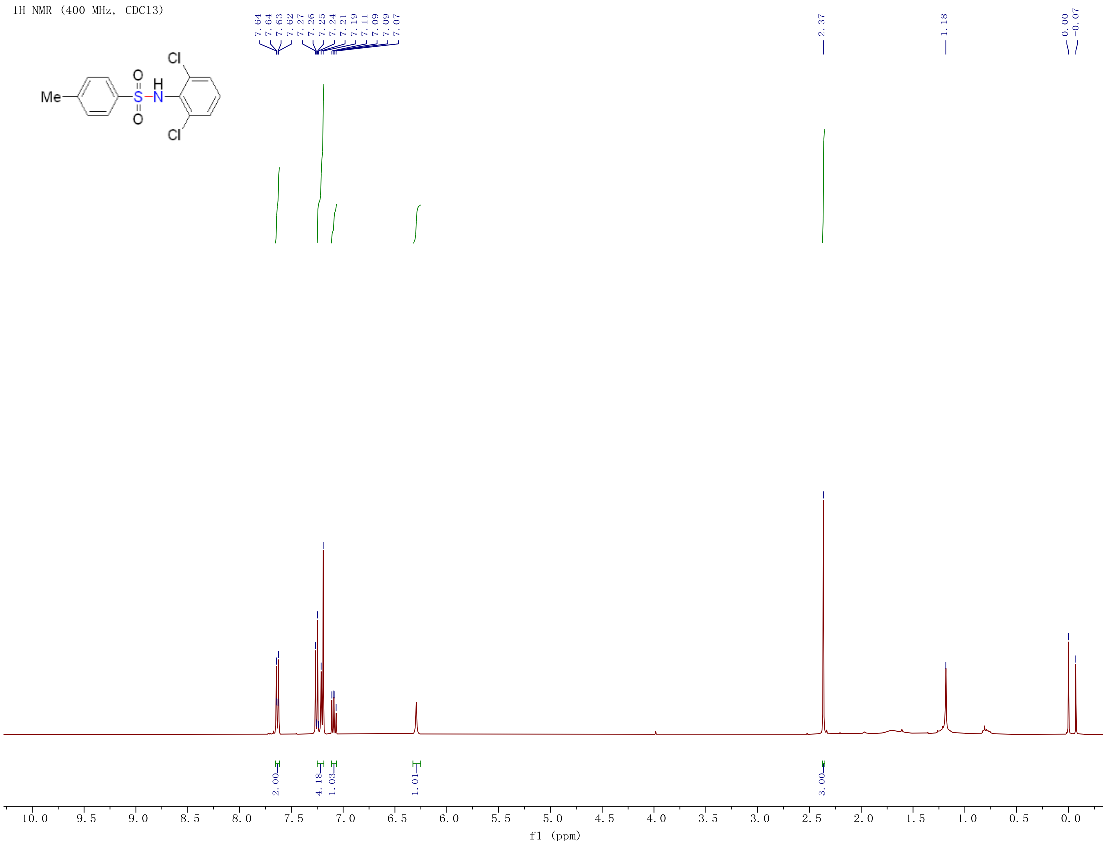

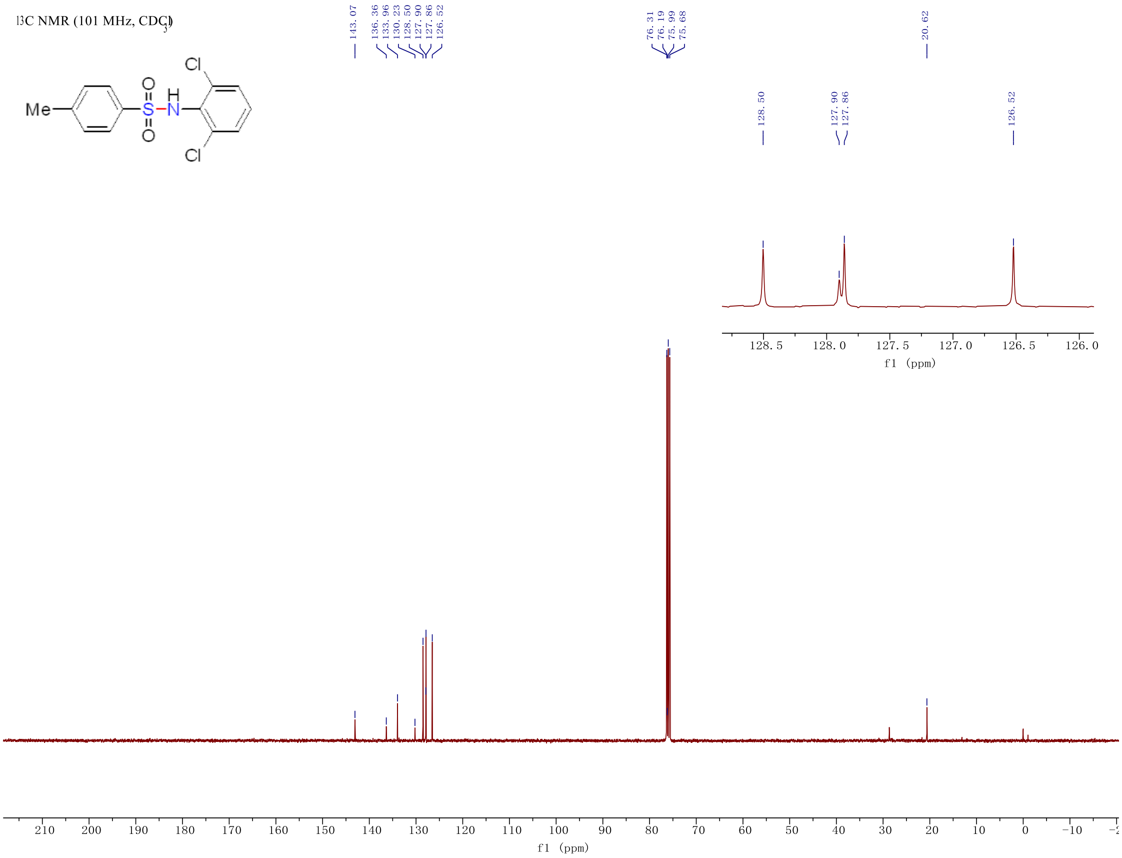

N-(*p*-tolyl)benzenesulfonamide (3m)

1H NMR (400 MHz, CDCl3)

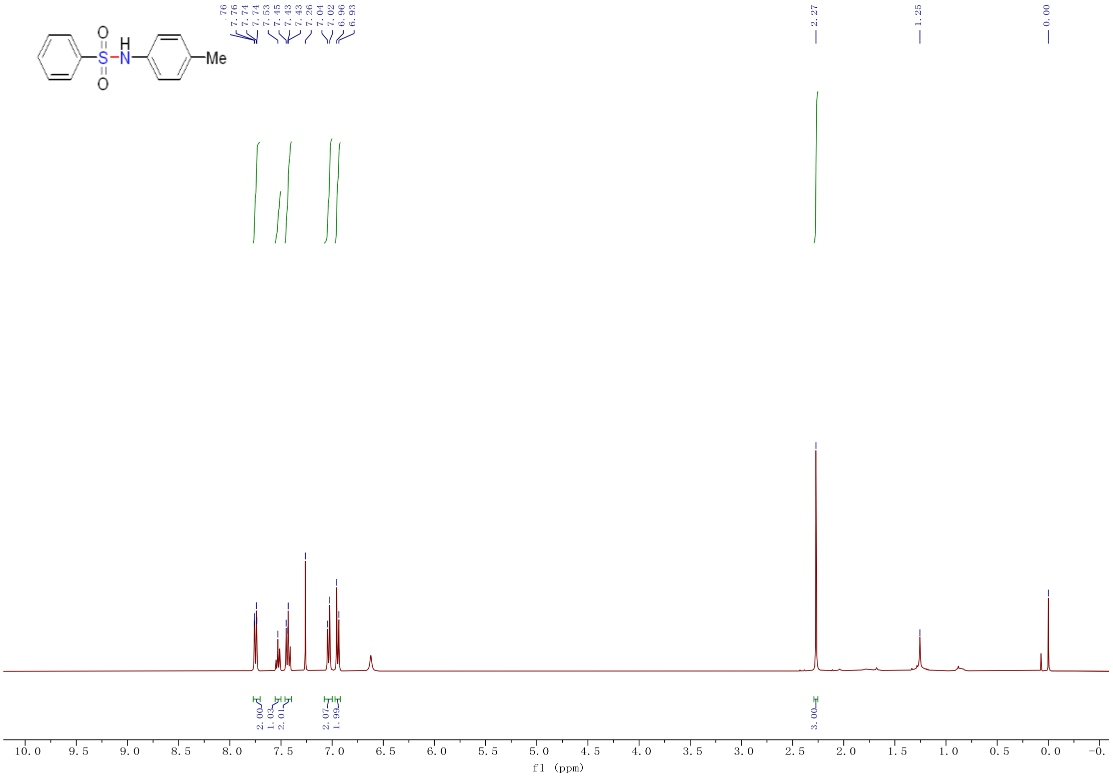

13C NMR (101 MHz, CDCl3)

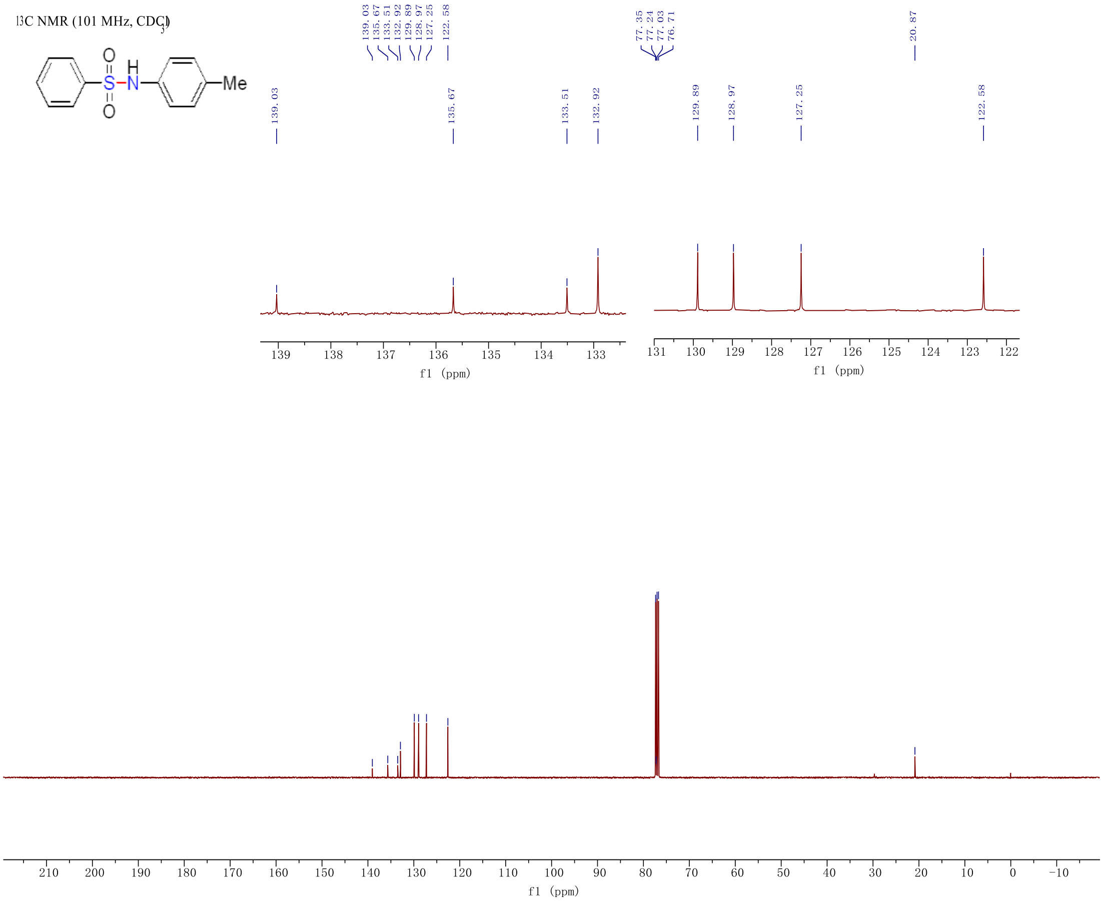

4-chloro-*N*-(*p*-tolyl)benzenesulfonamide (3n)

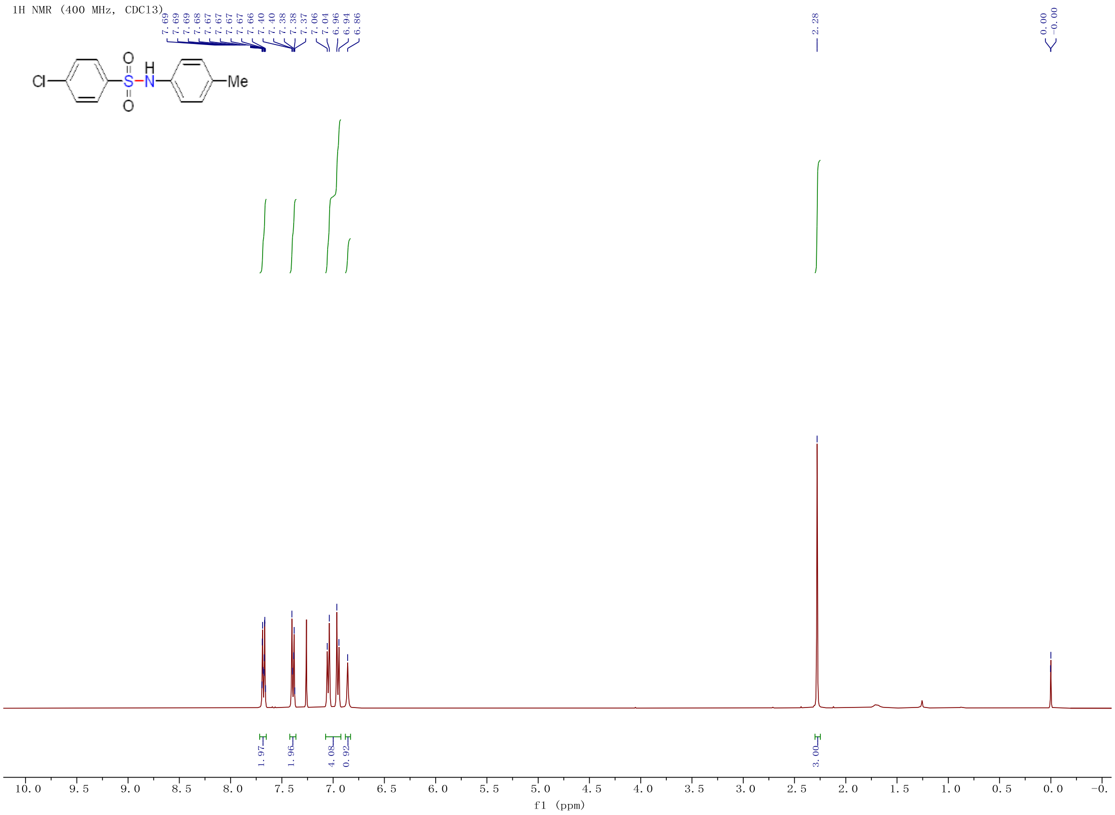

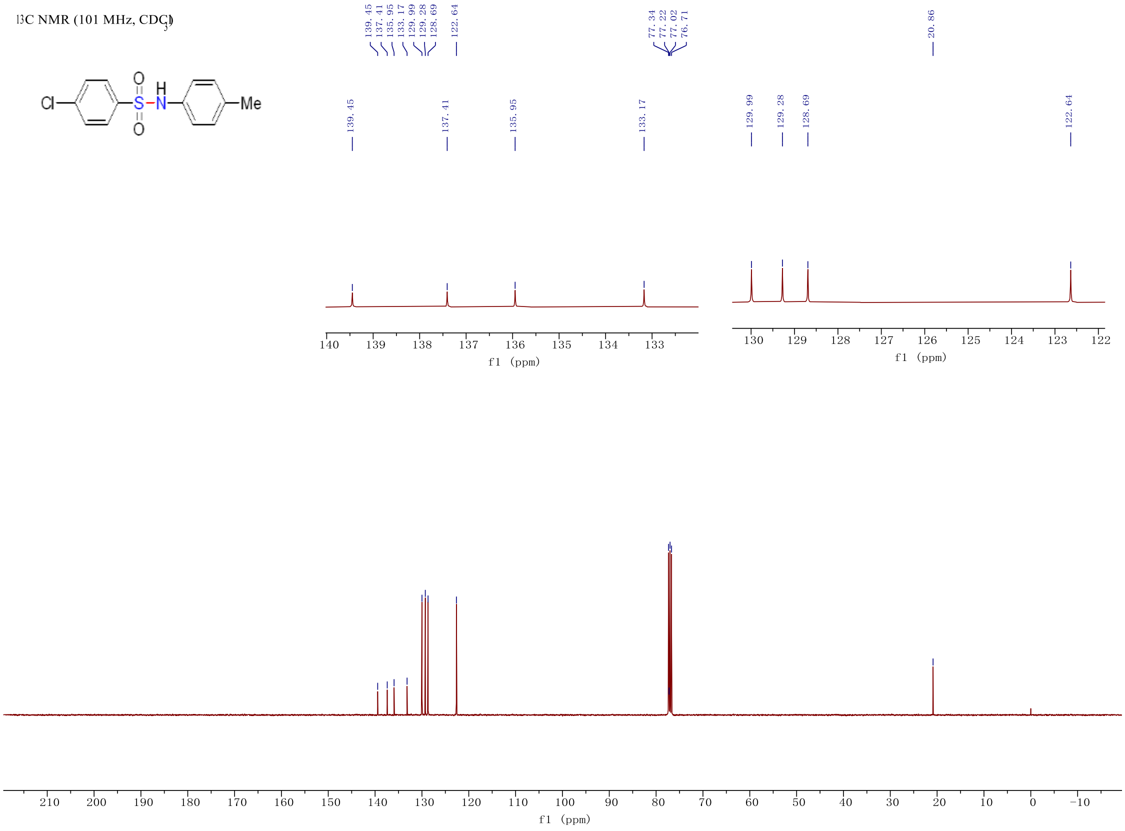

4-bromo-*N*-(*p*-tolyl)benzenesulfonamide (3o)

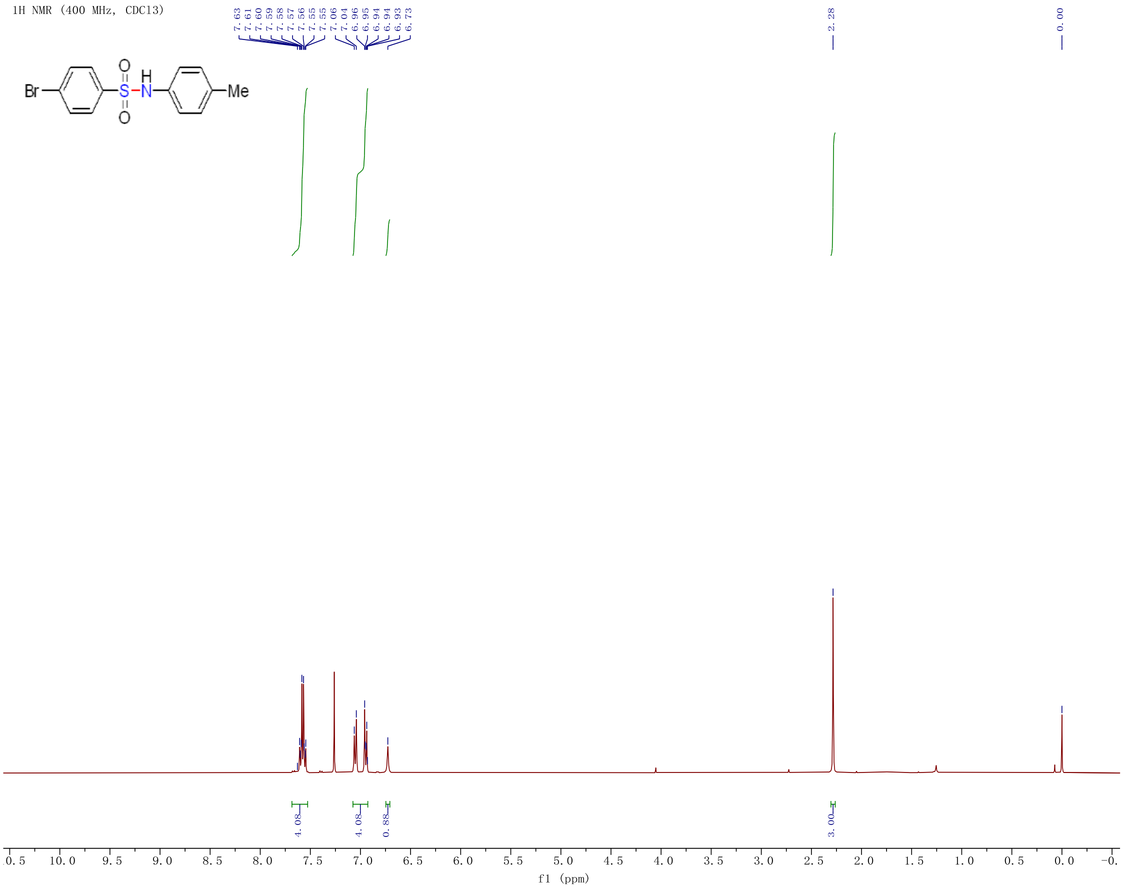

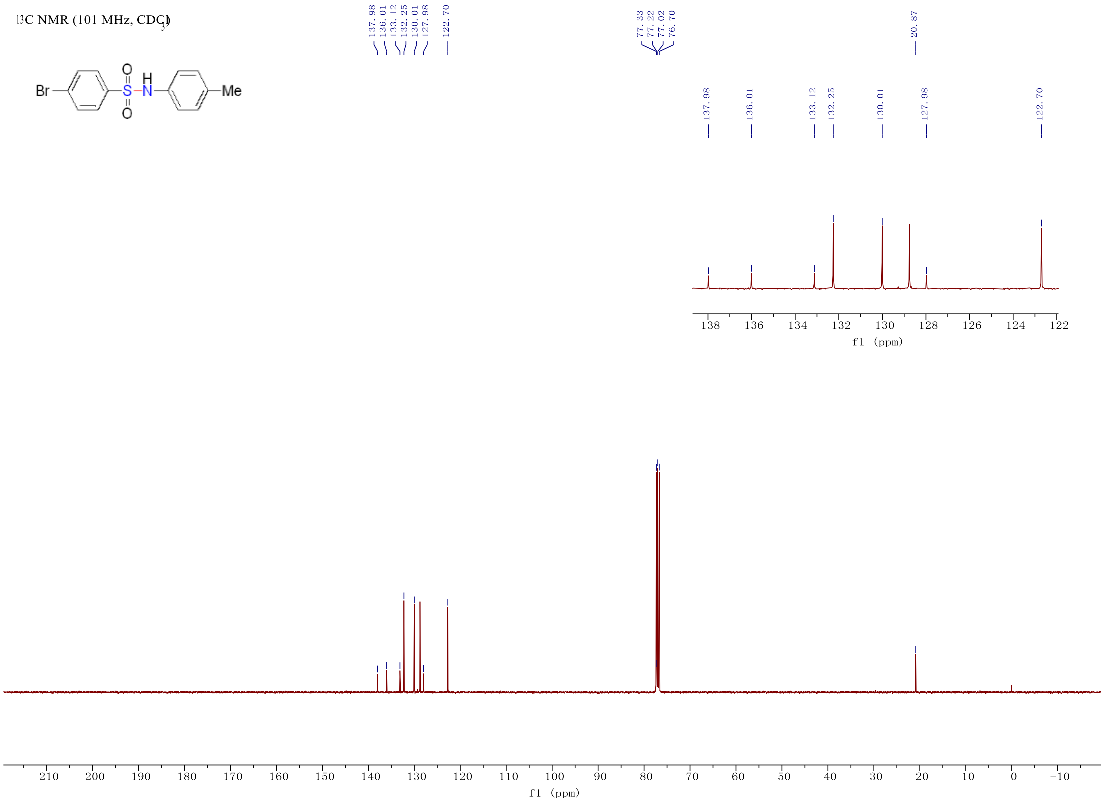

Supplement: Supplementary file 1 [file molecules-27-05539-s001.zip › molecules-1864864-supplementary.pdf]
